# Cox models with time‐varying covariates and partly‐interval censoring–A maximum penalised likelihood approach

**DOI:** 10.1002/sim.9645

**Published:** 2022-12-30

**Authors:** Annabel Webb, Jun Ma

**Affiliations:** ^1^ Department of Mathematics and Statistics Macquarie University Macquarie Park New South Wales Australia

**Keywords:** constrained optimization, partly‐interval censoring, penalized likelihood, time‐varying covariates

## Abstract

Time‐varying covariates can be important predictors when model based predictions are considered. A Cox model that includes time‐varying covariates is usually referred to as an extended Cox model. When only right censoring is presented in the observed survival times, the conventional partial likelihood method is still applicable to estimate the regression coefficients of an extended Cox model. However, if there are interval‐censored survival times, then the partial likelihood method is not directly available unless an imputation, such as the middle point imputation, is used to replaced the left‐ and interval‐censored data. However, such imputation methods are well known for causing biases. This paper considers fitting of the extended Cox models using the maximum penalised likelihood method allowing observed survival times to be partly interval censored, where a penalty function is used to regularise the baseline hazard estimate. We present simulation studies to demonstrate the performance of our proposed method, and illustrate our method with applications to two real datasets from medical research.

## 1 INTRODUCTION

The semi‐parametric Cox proportional hazards regression model^1^ is a widely used method in the analysis of survival (or time‐to‐event) data. Regression parameters in this model are typically estimated by maximising a partial likelihood (PL) function.^2^ Often, it may be of interest to investigate the relationship between survival times and predictor variables that cannot be assumed constant over the follow‐up time, and such predictors are called time‐varying covariates. A Cox model that contains time‐varying covariates is referred to as an extended Cox model. Methods for estimating regression coefficients of a Cox model with time‐varying covariates via the partial likelihood method have been proposed, notably by Reference 3; also see Reference 4. Some full likelihood based methods are also available, including References 5, 6, 7.

An important feature of survival data is the censoring scheme. Cox's original formulation of the proportional hazards model considered only directly observed event times and right‐censored observations, with the latter occurring when an event is not observed before the end of the follow‐up period. However, survival data sets may also contain left‐ and interval‐censored observations, where, respectively, the event time takes place prior to the first examination time and the event time is known to be between two examination times. We refer to a data set that may contain any combination of event times or right, left or interval censoring times as partly‐interval censored. If a dataset is partly‐interval censored, partial likelihood estimation of a Cox model is not available directly, and a number of full likelihood based methods have been proposed, such as References 8, 9, 10, 11. An alternative to these methods is to impute event times for interval‐ and left‐censored observations so that partial likelihood estimation becomes applicable, but this approach has been shown to produce biased estimates.^12^

Partly‐interval censored data with time‐varying covariates commonly arises in disease research or in observational data of long‐term illnesses. Monitoring the event status in these scenarios may take place at periodic follow‐up clinician appointments, leading to the presence of interval‐censored event times. Additionally, over the course of the follow‐up period, there may be repeated measurements of bio‐markers, or new treatments or surgeries introduced at various times, giving rise to time‐varying covariates. Frequently, the number and timing of periodic follow‐up appointments may differ across individuals in the data set (so‐called mixed case interval censored data^13^). Additionally, in some scenarios the monitoring of the event status and the monitoring of the time‐varying covariate may be independent, especially where diagnostic methods for the event of interest are expensive or invasive. For example, the event status (observed via the results of a diagnostic test) may be assessed at times $t_{1}$, $t_{2}$, and $t_{3}$, while a time‐varying covariate (such as the introduction of a new drug to a patient's treatment regimen) may change at none of these times. Clearly, there is a need for methods to fit time‐to‐event models to data with partly‐interval censored event times and time‐varying covariates which allow some flexibility in how data is observed.

Despite this, consideration of a Cox model with time‐varying covariates and partly‐interval censored observations has been limited, and previous methods may not be sufficiently flexible.^14^ considered a piece‐wise Cox model that can be fitted to interval censored data where a covariate is time dependent. However, their approach only allows for cases where the changing times for the time‐varying covariates are common across all individuals. Additionally, their parameter estimate may diverge in samples of size n≤200, and their method does not provide asymptotic variance estimates.^15^ considered a transformation model for interval censored data with time‐varying covariates, where the Cox model is a special case. This method allowed for mixed‐case interval censoring, but assumes that the event status and time‐varying covariate are monitored simultaneously, and additionally cannot incorporate directly observed event times in addition to interval censored times in the model.

In this paper, we develop a more flexible penalised likelihood approach for fitting a Cox model with time‐varying covariates and partly‐interval censored data. Our method allows the observation (or measurement) points for the time‐varying covariates to be different across individuals. The method provides both regression parameter estimates and a smooth estimate of the baseline hazard function, obtained via the use of a penalty term. Furthermore, we derive an asymptotic results that allow for inferences to be made not only on regression parameters, but also on survival quantities without relying on computationally intensive methods such as bootstrapping. To our knowledge, this is the first proposed method for fitting a time‐varying Cox model to interval‐censored data where a smooth estimate of the baseline hazard function is provided and asymptotic inferences on survival quantities are possible. We implement our method in a R package which is currently available on Github (see data availability statement), and eventually this package will be uploaded to The Comprehensive R Archive Network (CRAN).

The proposed method is situated within a wider body of research concerning penalised likelihood estimation of proportional hazard models.^11^, ^16^, ^17^, ^18^ Generally, this approach adds a roughness penalty term to the full log‐likelihood to produce a smooth baseline hazard function estimate. This maximum penalised likelihood (MPL) approach can easily incorporate partly‐interval censored data.^11^ For computing the MPL estimates, we approximate the baseline hazard function using a set of non‐negative basis functions. Then, in each iteration our approach employs a Newton step to update the regression coefficients and a multiplicative‐iterative step^19^ to estimate the approximated baseline hazard, and this estimate is guaranteed to be non‐negative. The Newton‐MI algorithm has been used for the MPL estimation of a variety of time‐to‐event models with partly‐interval censoring.^11^, ^20^, ^21^

In Section 2 we build the penalised log‐likelihood function from which the estimates on the regression coefficients and the approximated baseline hazard will be obtained. Section 3 develops the Newton‐MI algorithm for computing the constrained MPL estimates. Automatic selection of the smoothing parameter and some asymptotic results about the constrained MPL estimates will also be provided in this section. Simulation study results are presented in Section 4 and an application to two real data sets is contained in Section 5. Finally, some concluding remarks are give in Section 6.

## 2 EXTENDED COX MODEL AND PENALISED LIKELIHOOD

Let $T_{i}$ be the event time for subject i, where i=1,…,n. Under a partly interval censoring scheme, each subject will be associated with a random vector $Y_{i}={\left[Y_{i}^{L},Y_{i}^{R}\right]}^{⊤}$ such that $T_{i}\in [Y_{i}^{L},Y_{i}^{R}]$ and $Y_{i}^{L}\leq Y_{i}^{R}$. We denote the observed value of this random vector as $y_{i}={\left[y_{i}^{L},y_{i}^{R}\right]}^{⊤}$. Each $y_{i}$ corresponds to either an event time ($y_{i}^{L}=y_{i}^{R}=t_{i}$, $\delta_{i}=1$), a right censoring time ($y_{i}^{R}=\infty$, $\delta_{i}^{R}=1$), a left censoring time ($y_{i}^{L}=0$, $\delta_{i}^{L}=1$), or an interval censoring time ($y_{i}^{L}<y_{i}^{R}$, $\delta_{i}^{I}=1)$. Here $\delta_{i},\delta_{i}^{R},\delta_{i}^{L}$ and $\delta_{i}^{I}$ are indicators for respectively event time and right, left and interval censoring times. Note that these indicators are employed mainly to simplify the likelihood expression. Throughout this article, we assume independent interval censoring given the covariates, meaning $P(T_{i}\leq t|Y_{i}^{L}=y_{i}^{L},Y_{i}^{R}=y_{i}^{R},Y_{i}^{L}\leq T_{i}<Y_{i}^{R})=P(T_{i}\leq t|y_{i}^{L}\leq T_{i}<y_{i}^{R})$ (see, eg, Chap. 1 of References 22 and 23).

In this paper, we consider how to fit the following extended Cox model where time‐varying covariates are present:

(1) $$h_{i}(t)=h_{0}(t)e^{x_{i}^{⊤}\beta +z_{i}{\left(t\right)}^{⊤}\gamma },$$

where the covariates for subject i are separated into p baseline (time‐fixed) covariates $x_{i}={\left[x_{i1},\ldots ,x_{ip}\right]}^{⊤}$ and q time‐varying covariates $z_{i}(t)={\left[z_{i1}(t),\ldots ,z_{iq}(t)\right]}^{⊤}$, β and γ are vectors for regression coefficients for time‐fixed and time‐varying covariates respectively and $h_{0}(t)$ is the non‐parametric baseline hazard function, which must be non‐negative. We can re‐express the hazard function (1) as

(2) $$h_{i}(t)=h_{0i}^{*}(t)e^{x_{i}^{⊤}\beta }$$

where $h_{0i}^{*}(t)=h_{0}(t)e^{z_{i}{\left(t\right)}^{⊤}\gamma }$.

The proposed method estimates the infinite dimensional parameter $h_{0}(t)$ by simplifying it to a finite dimensional parameter via the use of non‐negative basis function approximation. That is, we approximate $h_{0}(t)$ by

(3) $$h_{0}(t)=\overset{m}{\underset{u=1}{\sum }}\theta_{u}\psi_{u}(t)$$

where $\psi_{u}(t)\geq 0$ is the $u^{th}$ non‐negative basis function and $\theta =[\theta_{1},\ldots ,\theta_{m}]$ is a vector of coefficients. We have the following two comments about this approximation.

1. All $\theta_{u}$ are restrained non‐negative ($\theta_{u}\geq 0$) so that $h_{0}(t)\geq 0$ is guaranteed.
2. The number of basis functions m can vary with the sample size n and thus this approximation does not result in a parametric approach.

Correspondingly, the approximate cumulative baseline hazard is $H_{0}(t)=\sum_{u=1}^{m}\theta_{u}\Psi_{u}(t)$, where $\Psi_{u}(t)=\int_{0}^{t}\psi_{u}(s)ds$ is the $u^{th}$ cumulative basis function. The cumulative hazard for subject i, corresponding to (2), can be expressed as

(4) $$H_{i}(t)=H_{0i}^{*}(t)e^{x_{i}^{⊤}\beta },$$

where $H_{0i}^{*}(t)=\sum_{u}\theta_{u}\Psi_{ui}^{*}(t)$ and $\Psi_{ui}^{*}(t)=\int_{0}^{t}\psi_{u}(s)e^{z_{i}{\left(s\right)}^{⊤}\gamma }ds.$ In this paper we will consider the use of cubic M‐splines^24^ as the basis functions. These spline functions are readily available in R, for example in the package splines2.^25^

In practice, it is often sufficient to treat the time‐varying covariates $z_{ib}(t)$, b=1,…,q, as discrete functions of time, such that any $z_{ib}(t)$ remains a constant, denoted as $z_{iab}$ below, in each of the $n_{i}$ time intervals for subject i. That is, we assume

(5) $$z_{ib}(t)=z_{iab}\;I(t_{ia}\leq t<t_{i,a+1}),$$

where I is an indicator function, $a=0,\ldots ,n_{i}-1$, and the $t_{ia}$ can be considered the changing points of $z_{ib}(t)$. Without loss of generality, we can say that $t_{i0}=0$ and $t_{n_{i}}$ is the maximum value of $\left[y_{i}^{L},y_{i}^{R}\right]$ that is <∞, which for convenience is denoted by $y_{i}$. Therefore, each $z_{ib}(t)$ is a piece‐wise constant with $n_{i}$ pieces over $\left[0,\;y_{i}\right]$. For each individual, the set of observed information $\left(y_{i}^{L},y_{i}^{R},\delta_{i},\delta_{i}^{R},\delta_{i}^{L},\delta_{i}^{I},x_{i},z_{i}(t_{ia})\right)$ is available. We assume that for each i, $n_{i}$ and the times $t_{ia}$ for $a=0,\ldots ,n_{i}-1$ are known directly.

Partly interval censored data, in practice, may arise from contexts where individuals are subject to a series of examination times at which their event status is assessed (doctor's appointments, blood tests, scans, biopsies, etc.). At these event status assessment times, the value of a time‐varying covariate may or may not also be observed. This model makes no assumption about the relationship between the timing of the changing points in $z_{ib}(t)$ and the times at which an individual's event status is assessed. Specifically, there may or may not be a value of a such that $t_{ia}=t_{i}^{L}$. However, the value of $z_{ib}(t_{i}^{L})$ can be imputed when needed by finding $z_{iab}(t_{i}^{L})I(t_{ia}\leq t_{i}^{L}<t_{i,a+1})$ for $a=1,\ldots ,n_{i}$.

Since $z_{ib}(t)$ are now discrete, the calculation of the integral in the expression for $\Psi_{ui}^{*}(t)$ is simplified. For $t\in (t_{i,d-1},t_{i,d}]$, we have

(6) $$\Psi_{ui}^{*}(t)=\overset{d-1}{\underset{a=0}{\sum }}[\Psi_{u}(t_{i,a+1})-\Psi_{u}(t_{ia})]e^{z_{ia}^{⊤}\gamma },$$

where $z_{ia}={\left[z_{ia1},\ldots ,z_{iaq}\right]}^{⊤}$, and therefore

(7) $$H_{0i}^{*}(t)=\overset{d-1}{\underset{a=0}{\sum }}[H_{0}(t_{i,a+1})-H_{0}(t_{ia})]e^{z_{ia}^{⊤}\gamma }.$$

We specify that $\Psi_{u}(t_{i0})=0$ and $H_{0}(t_{i0})=0$ for all i and u.

The log‐likelihood function for independently observed partly interval‐censored survival time is

(8) $$\begin{array}{ll} l(\beta ,\gamma ,\theta ) & =\overset{n}{\underset{i=1}{\sum }}\delta_{i}(\ln h_{0}(y_{i})+x_{i}^{⊤}\beta +z_{iy_{i}}^{⊤}\gamma -H_{i}(y_{i}))-\delta_{i}^{R}H(y_{i}^{L})\\ & \;+\delta_{i}^{L}\ln(1-S(y_{i}^{R}))+\delta_{i}^{I}\ln(S(y_{i}^{L})-S(y_{i}^{R})). \end{array}$$

Let $\eta ={\left[\beta^{⊤},\gamma^{⊤},\theta^{⊤}\right]}^{⊤}$. We wish to estimate η by employing the log‐likelihood (8). To obtain a smooth estimate for the baseline hazard function $h_{0}(t)$, and to a lesser extend to make the final estimates less sensitive to the number and location of the knots, this log‐likelihood is penalised, giving

(9) Φ(η)=l(η)−λJ(θ),

where J(θ) is a roughness penalty and λ≥0 is a smoothing parameter. In this paper we adopt a quadratic roughness penalty function given by $\int h_{0}^{\prime \prime }{\left(v\right)}^{2}dv$. Given the approximation of $h_{0}(t)$ in (3), The roughness penalty can be expressed as $J(\theta )=\theta^{⊤}R\theta$, where R is an m×m matrix with the ${\left(u,v\right)}^{th}$ element given by $r_{u,v}=\int \psi_{u}^{\prime \prime }(t)\psi_{v}^{\prime \prime }(t)dt$.

## 3 MAXIMUM PENALISED LIKELIHOOD ESTIMATION

### 3.1 Constrained optimization calculation

A constrained optimizing algorithm will be used to obtain estimates for η while ensuring that all $\theta_{u}\geq 0$ so that $h_{0}(t)$ is guaranteed to be non‐negative. The maximum penalised likelihood estimate of η, denoted by $\hat{\eta }$ is given by

(10) $$\hat{\eta }=\underset{\theta \geq 0}{\max}\Phi (\eta ),$$

where θ≥0 (meaning each element of θ is non‐negative) is the constraint we need to impose. The constrained optimal solution has the following Karush‐Kuhn‐Tucker (KKT) conditions:

$$\begin{array}{ll} & \frac{\partial \Phi (\eta )}{\partial \beta }=0,\;\;\frac{\partial \Phi (\eta )}{\partial \gamma }=0;\;\\ & \frac{\partial \Phi (\eta )}{\partial \theta_{u}}=0\;\text{if}\;\theta_{u}>0,\;\;\frac{\partial \Phi (\eta )}{\partial \theta_{u}}<0\;\text{if}\;\theta_{u}=0,\; \end{array}$$

where u=1,…,m. These conditions are solved iteratively using an algorithm similar to the Newton‐MI algorithm of Reference 11. This algorithm requires the score vector and the Hessian matrix for β and γ, but for θ it only requires the score vector. Its fundamental idea is described as the following. Let $\beta^{\left(k\right)}$, $\gamma^{\left(k\right)}$ and $\theta^{\left(k\right)}$ be, respectively, the values of the estimates for β, γ and θ at iteration k. Iteration k+1 of our algorithm is obtained in an alternating three‐step process:

1. Compute $\beta^{\left(k+1\right)}$ so that $\Phi (\beta^{\left(k+1\right)},\gamma^{\left(k\right)},\theta^{\left(k\right)})\geq \Phi (\beta^{\left(k\right)},\gamma^{\left(k\right)},\theta^{\left(k\right)})$.
2. Compute $\gamma^{\left(k+1\right)}$ so that $\Phi (\beta^{\left(k+1\right)},\gamma^{\left(k+1\right)},\theta^{\left(k\right)})\geq \Phi (\beta^{\left(k+1\right)},\gamma^{\left(k\right)},\theta^{\left(k\right)})$.
3. Compute $\theta^{\left(k+1\right)}$ so that $\Phi (\beta^{\left(k+1\right)},\gamma^{\left(k+1\right)},\theta^{\left(k+1\right)})\geq \Phi (\beta^{\left(k+1\right)},\gamma^{\left(k+1\right)},\theta^{\left(k\right)})$.

These conditions ensure that the penalised likelihood Φ(η) is maximised at the end of the iterative process.

To update β in Step 1, we employ a Newton algorithm with a line search step size. Starting with $\beta^{\left(k\right)}$ and a step size $\omega_{1}^{\left(k\right)}$, we have

(11) $$\beta^{\left(k+1\right)}=\beta^{\left(k\right)}+\omega_{1}^{\left(k\right)}{\left(X^{⊤}A^{\left(k\right)}X\right)}^{-1}X^{⊤}(\delta -C^{\left(k\right)}1_{n}).$$

Here, X is an n×p model matrix of time‐fixed covariates, δ is an n‐vector with elements equal to 1 for event times and 0 for others and $1_{n}$ is a vector of 1's with length n and both C and A are n×n diagonal matrices with diagonal vectors c and a respectively. Vectors c and a are specified in the Supplementary Material of this paper. From the expressions for vectors c and a, if a data set contains only right‐censoring and event times (ie, $\delta_{i}^{L}=\delta_{i}^{I}=0$), then we will have A=C.

Updating γ in Step 2 is similarly achieved using a Newton algorithm with a line search step size but a totally different model matrix as explicated below. Let $N=\sum_{i=1}^{n}n_{i}$ and $Z_{i}={\left(z_{i1},\ldots ,z_{in_{i}}\right)}^{⊤}$, where vectors $z_{ia}$ were defined after equation (7). Define $Z={\left(Z_{1}^{⊤},\ldots ,Z_{n}^{⊤}\right)}^{⊤}$. Matrix Z (with the dimension N×q) can be viewed as a long formatted model matrix for the time varying covariates. Starting with a current estimate $\gamma^{\left(k\right)}$ and a step size $\omega_{2}^{\left(k\right)}$, γ is updated according to

(12) $$\gamma^{\left(k+1\right)}=\gamma^{\left(k\right)}+\omega_{2}^{\left(k\right)}{\left(Z^{⊤}B^{\left(k\right)}Z\right)}^{-1}Z^{⊤}(\epsilon^{E}-E^{\left(k\right)}1_{N})$$

where specifications for vector $\epsilon^{E}$ and block diagonal matrices B and E are provided in the Supplementary Material.

Finally, for updating θ in Step 3, we use the multiplicative‐iterative algorithm (eg, Reference 19) so that the estimate of θ is guaranteed to be non‐negative. This algorithm can also be expressed as a gradient algorithm.^19^ From a current estimate $\theta^{\left(k\right)}$ and a line search step size $\omega_{3}^{\left(k\right)}$, we update θ using

(13) $$\theta^{\left(k+1\right)}=\theta^{\left(k\right)}+\omega_{3}^{\left(k\right)}{\left[S^{\left(k\right)}\right]}^{-1}g_{\theta }^{\left(k\right)},$$

where S is a diagonal matrix involving only the first derivative of θ and $g_{\theta }$ is the gradient vector for θ. Formulas for S and $g_{\theta }$ can be found in the Supplementary Material.

We comment that all three line searches for $\omega_{1}$, $\omega_{2}$ and $\omega_{3}$ can be conducted easily using, for instance, Armijo's method (eg, Reference 26).

### 3.2 Automatic smoothing parameter selection

Automatic selection of the smoothing parameter is important for our proposed method, as a poor specification of the smoothing parameter may lead to a sub‐optimal solution. In this paper, we adopt a marginal likelihood method for automatic selection of the smoothing parameter similar to, for example, References 27 and 11. This method builds on the idea of relating the penalty function of θ to a multivariate normal prior distribution $\theta ∼N(0_{m\times 1},\sigma^{2}R^{-1})$, where $\sigma^{2}=1/2\lambda$. Under this setting, the log‐posterior is

(14) $$l_{p}(\beta ,\gamma ,\theta )=l(\beta ,\gamma ,\theta )-\frac{m}{2}\ln\sigma_{\theta }^{2}-\frac{1}{2\sigma^{2}}\theta^{⊤}R\theta .$$

The marginal likelihood method demands a marginal likelihood for $\sigma^{2}$, defined as the function after integration out β,γ,θ from the posterior in (14). Due to the possible high‐dimensional nature of β,γ,θ, direct evaluation of this integral can be difficult. As such, we can approximate the integral using Laplace's method, and this leads to the following approximated log‐marginal likelihood:

(15) $$l_{m}(\sigma^{2})\approx -\frac{m}{2}\log\sigma^{2}+l(\hat{\beta },\hat{\gamma },\hat{\theta })-\frac{1}{2\sigma^{2}}{\hat{\theta }}^{⊤}R\hat{\theta }-\frac{1}{2}\log|\hat{G}+Q_{\sigma^{2}}|,$$

where $\hat{\beta },\hat{\gamma }$ and $\hat{\theta }$ denote the MPL estimates when $\sigma^{2}$ is known, $\hat{G}$ is the negative Hessian matrix from l(β,γ,θ) evaluated at the MPL estimates $\hat{\beta }$, $\hat{\gamma }$ and $\hat{\theta }$, and

$$Q_{\sigma^{2}}=\left[\begin{array}{ccc} 0_{p\times p} & 0_{p\times q} & 0_{p\times m}\\ 0_{q\times p} & 0_{q\times q} & 0_{q\times m}\\ 0_{m\times p} & 0_{m\times q} & \frac{1}{\sigma^{2}}R \end{array}\right],$$

where 0 denotes a matrix with its elements are all zero. The solution to $\sigma^{2}$ maximising (15), denoted by ${\hat{\sigma }}^{2}$, satisfies

(16) $${\hat{\sigma }}^{2}=\frac{{\hat{\theta }}^{⊤}R\hat{\theta }}{m-\nu },$$

where $\nu =\text{tr}\{{\left(\hat{G}+Q_{{\hat{\sigma }}^{2}}\right)}^{-1}Q_{{\hat{\sigma }}^{2}}\}$. Here, m−ν can be considered as equivalent to the model degrees of freedom. The expression in (16) for $\sigma^{2}$ allows for the development of an iterative procedure for estimation of β, γ, θ and $\sigma^{2}$ with two steps. Firstly, with a current $\sigma^{2}$ estimate, the corresponding MPL estimates for β, γ and θ are obtained. Then, $\sigma^{2}$ is updated using these obtained MPL estimates of β, γ and θ on the right hand side of (16). These two steps are repeated until ν is stabilised, for instance when the difference between two consecutive ν values is less than 1.

### 3.3 Asymptotic properties

In this part of the section, we state the asymptotic consistency and the large sample normality results for the MPL estimates of β,γ and θ. The assumptions needed for these results can be found in the Supplementary Material. The large sample normality result is particularly helpful as it can be used for inference tasks on regression parameters and survival quantities when the sample size is large, avoiding computationally intensive (thus time‐consuming) methods such as bootstrapping.

We first provide the asymptotic consistency result in Theorem 1. Let the true β, γ and $h_{0}(t)$ be denoted by $\beta_{0}$, $\gamma_{0}$ and $h_{00}(t)$ respectively. Let a and b be the minimum and maximum of all the observed survival times respectively, including interval censoring but excluding 0 and ∞.

Theorem 1 *Assume that conditions (A1)–(A4) in the Supplementary Material hold. Assume that* $h_{0}(t)$ *is bounded and has some number* r≥1 *derivatives over the interval* [a,b] *. Assume that* $m=n^{v}$ *, where* 0<v<1 *. Then, when* n→∞,

*1*. $||\hat{\beta }-\beta_{0}||\to 0$ *almost surely*,
*2*. $||\hat{\gamma }-\gamma_{0}||\to 0$ *almost surely, and*
*3*. $\sup_{t\in [a,b]}|{ĥ}_{0}(t)-h_{00}(t)|\to 0$ *almost surely*.

Next, we will provide in Theorem 2 the large sample normality result for the MPL estimates. This result provides an efficient way for making inferences. In this result, m is allowed to vary with n. In practice, we adopt a simple guideline for selecting m: $m=n_{0}^{1/3}$, where $n_{0}$ is the non‐right censored sample size. The simulation results in Section 4 demonstrate this large sample result is in general accurate in terms of bias, standard error and coverage probability.

A difficult issue here is that some elements of θ≥0 can be actively constrained. Active constraints are particularly likely to occur when there are more knots selected than is necessary. Ignoring active constraints may lead to negative variance for some of the MPL estimates. Recall $\eta ={\left[\beta^{⊤},\gamma^{⊤},\theta^{⊤}\right]}^{⊤}$, so the length of η is p+q+m. Let the MPL estimate of η be denoted by $\hat{\eta }$, and let $\eta_{0}$ be the true value of the parameter vector. Without loss of generality, we assume that the first r of θ elements are 0, that is, they are actively constrained. According to this assumption we define

(17) $$U=[0_{(m-r+p+q)\times r},I_{\left(m-r+p+q)\times (m-r+p+q\right)}]$$

where I represents an identity matrix. Theorem 2 states the large sample normality result. We refer to Reference 11 for detailed proofs of this result and the assumptions required for this result are included in the Supplementary Material.

Theorem 2 *Assume the first* r *elements of the constraint* θ≥0 *are active. Define matrix* U *as in (* *17* *) above. Assume that assumptions B1‐B5 in the Supplementary Material hold and that the smoothing parameter* λ *satisfies* λ≪n *. Define*

$$A{\left(\hat{\eta }\right)}^{-1}=U{\left(U^{T}\left(-\frac{\partial^{2}l(\hat{\eta })}{\partial \eta \partial \eta^{T}}+\lambda \frac{\partial^{2}J(\hat{\eta })}{\partial \eta \partial \eta^{T}}\right)U\right)}^{-1}U^{T}$$

*Then, when* n *is large, the distribution for the MPL estimate* $\hat{\eta }-\eta_{0}$ *can be approximated by a multivariate normal distribution having mean zero and covariance matrix*

$$\hat{\mathrm{var}}(\hat{\eta })=A{\left(\hat{\eta }\right)}^{-1}\left(-\frac{\partial^{2}l(\hat{\eta })}{\partial \eta \partial \eta^{T}}\right)A{\left(\hat{\eta }\right)}^{-1}$$

To implement the result of Theorem 2, we require a method for identifying active constraints in practice. The method proposed here closely follows that of Reference 11. Active constraints can be identified by inspecting both the value of ${\hat{\theta }}_{u}$ and the corresponding gradient for each u. After the Newton‐MI algorithm has reached convergence, some ${\hat{\theta }}_{u}$ may be exactly zero with negative gradients, and thus clearly they are active constraints. Furthermore, there may be some ${\hat{\theta }}_{u}$ that are very close to, but not exactly, zero. For these ${\hat{\theta }}_{u}$, a corresponding negative gradient value is indicative that they are also subject to an active constraint. In practice, active constraints are defined where, for a given u, ${\hat{\theta }}_{u}<10^{-2}$ and the corresponding gradient is less than −ε where ε is a positive threshold value such as $10^{-2}$.

## 4 SIMULATION STUDIES

To evaluate the proposed method, we carried out two simulation studies. Study 1 considered merely right‐censored survival data and in this study we compared our proposed method with the Cox partial likelihood method, fitted using the coxph(·) function in the R survival package.^28^ Study 2 evaluated the performance of our proposed method when fitted to partly‐interval censored data. In Study 2, two different scenarios were considered, and one scenario allowed for comparison between the proposed MPL method and the transformation model (TM) method proposed by Reference 15, which to our knowledge is the only publicly available R package for fitting a Cox model to interval censored survival data that has time‐varying covariates. In both studies, we evaluated the estimates of the regression parameter and baseline survival function through their performance on bias, asymptotic standard error (comparing with Monte Carlo standard error) and coverage probability, where 500 simulated data sets were used to perform these calculations. Some simulation results will be provided in this paper; others will be included in the Supplementary Material. Before presenting the simulation results, we first explain survival data generation in these two studies.

### 4.1 Data generation strategy

In Study 1, independent survival times $t_{i}$ were simulated from the model given in (1) using a Gompertz distribution for the baseline hazard so that $h_{0}(t)=0.5e^{0.2t}$, or using an exponential distribution for the baseline hazard so that $h_{0}(t)=t$. The simulation results from the Gompertz baseline hazard are reported in this paper, and the results from the exponential baseline hazard are given in the Supplementary Material. In this study, two time‐fixed (baseline) covariates $X_{1}$ and $X_{2}$ were drawn, respectively, from a Bernoulli distribution Bern(0.5) and a uniform distribution Unif[0,1]. One time‐varying covariate $Z_{1}$ was used in this model where $Z_{1}$ was allowed to change a maximum of once across the follow‐up period: the value of $Z_{1}$ was set to 0 at baseline for all individuals and was able to change to 1 at a random changing time. Changing times for $Z_{1}$ were drawn from a uniform $\text{Unif}[\tau_{1},\tau_{2}]$ distribution, where the values of $\tau_{1}$ and $\tau_{2}$ could be adjusted to control the proportion of the sample where a change in $Z_{1}$ was observed before the event/censoring time. We selected the regression parameters for $X_{1}$ and $X_{2}$ to be $\beta_{1}=1$ and $\beta_{2}=-0.5$ and for $Z_{1}$ to be $\gamma_{1}=-1$. We generated random survival times from this time‐varying covariates Cox model model using the approach laid out in.^29^ Study 1 considered only right censoring, with right censoring times $c_{i}$ drawn from the uniform distribution $\text{Unif}[b_{1},b_{2}]$, where the parameters $b_{1}$ and $b_{2}$ could be adjusted to control the amount of right censoring in the sample. Two sample sizes, n=200 and n=1000, were used in the simulation studies. Two levels of event proportions, $\pi^{E}=0.3$ and $\pi^{E}=0.7$, were considered.

Study 2 considered partly‐interval censoring under two different sets of assumptions (thus two scenarios) about the follow‐up observations. The reason for this design was that we wished to compare our method with the transformation model (TM) method proposed by Reference 15, but the latter method only accepts left‐, right‐ and interval‐censored times (so that exact event times are not allowed). In the first part of Study 2 (Scenario 1), we generated the observed survival times that include event times and right‐, left‐ and/or interval‐censoring times. In the second part (Scenario 2), the simulated survival times were either left‐, right‐ or interval‐censored, and did not contain exact event times. Apart from the observed survival data difference between these two scenarios, their assessment times for the time‐varying covariates were also different (see below) as the TM R package requires that a record on survival status is also made at each assessment time of the time‐varying covariates. Details of the two scenarios of Study 2 are provided below.

Scenario 1: For this scenario, we simulated independent survival times $y_{i}$ from the model in (1) with either a Weibull baseline hazard function, so that $h_{0}(t)=3t^{2}$, or a log‐logistic baseline hazard function, so that $h_{0}(t)=4.5t/(1+t^{2})$. The model contained two time‐fixed covariates $X_{1}$ and $X_{2}$ and one time‐varying covariate $Z_{1}$, where all these covariates values were generated in the same way as Study 1 and the regression coefficients were also the same as Study 1. The results for the Weibull hazard will be reported in this paper, while for the log‐logistic hazard the results are in the Supplementary Material. After each event time $t_{i}$ was obtained, an observed survival time interval ($y_{i}^{L}$, $y_{i}^{R}$) was generated according to the following specifications:
$$y_{i}^{L}=t_{i}^{I(U_{i}^{E}<\pi^{E})}{\left(\alpha_{L}U_{i}^{L}\right)}^{I(\pi^{E}<U_{i}^{E},\alpha_{L}U_{i}^{L}\leq t_{i}\leq \alpha_{R}U_{i}^{R})}{\left(\alpha_{R}U_{i}^{R}\right)}^{I(\pi^{E}\leq U_{i}^{E},\alpha_{R}U_{i}^{R}<t_{i})}0^{I(\pi^{E}\leq U_{i}^{E},t_{i}<\alpha_{L}U_{i}^{L})}$$
  and
$$y_{i}^{R}=t_{i}^{I(U_{i}^{E}<\pi^{E})}{\left(\alpha_{L}U_{i}^{L}\right)}^{I(\pi^{E}\leq U_{i}^{E},t_{i}<\alpha_{L}U_{i}^{L})}{\left(\alpha_{R}U_{i}^{R}\right)}^{I(\pi^{E}\leq U_{i}^{E},\alpha_{L}U_{i}^{L}\leq t_{i}\leq \alpha_{R}U_{i}^{R})}\infty^{I(\pi^{E}\leq U_{i}^{E},\alpha_{R}U_{i}^{R}<t_{i})}$$
  where $\pi^{E}$ denotes the event proportion, $U_{i}^{E}$, $U_{i}^{L}$ and $U_{i}^{R}$ denote independent standard uniform variables for generating event times and interval censoring times, and $\alpha_{L}$ and $\alpha_{R}$ are scalars that define the width of the censoring intervals and therefore they can affect the proportions of left, right and interval censoring. Function I(·) in the $y_{i}^{L}$ and $y_{i}^{R}$ expressions represents an indicator function. Again, two sample sizes, n=200 and n=1000, were used in the simulation studies, and two levels of event proportions, $\pi^{E}=0.3$ and $\pi^{E}=0.7$, were considered. Note that in general we selected values of $\tau_{1}$ and $\tau_{2}$ (the values defining the timing of the change points in the time‐varying covariate) so that the distribution of the values $Z_{1}=0$ and $Z_{1}=1$ were approximately balanced in the sample. To investigate the effect on model estimation when these value were unbalanced, we performed a small sub‐study where the timing of the change point was pushed towards the end of the follow‐up period, so that only a small proportion of each sample ever had a value of $Z_{1}=1$, and these results are presented in the Supplementary Material.
Scenario 2: For the second scenario, we generated independent event times $t_{i}$, now using only a Weibull distribution. All the simulated survival times were either left‐, interval‐ or right‐censored. Again, two sample sizes of n=200 and n=1000 were considered for this scenario. We investigated three conditions for the covariates used in the model as specified below.
  Condition 1 Under this condition, two time‐fixed covariates $X_{1}$ and $X_{2}$ and one time‐varying covariate $Z_{1}$ were used as in Scenario 1, but now $Z_{1}$ was allowed to change between 0 and 1 up to three times for each i. This condition had true parameter values set at $\beta_{1}=0.5$, $\beta_{2}=-1$ and $\gamma_{1}=-0.5$.
  Condition 2 This condition used two time‐fixed covariates $X_{1}$ and $X_{2}$ and two discrete time‐varying covariates $Z_{1}$ and $Z_{2}$, where $Z_{1}$ could change between 0 and 1 a maximum of once, and $Z_{2}$ could change between 0 and 1 a maximum of twice. This condition had true parameter values set at $\beta_{1}=0.5$, $\beta_{2}=-1$, $\gamma_{1}=-0.5$ and $\gamma_{2}=1$. For these first two conditions, the intervals between changing times for the time‐varying covariates were generated using Unif[$\tau_{1}$, $\tau_{2}$]
  Condition 3 This condition used one time‐fixed covariate $X_{1}$ and one continuous time‐varying covariate $Z_{1}$, where for each i the value the time‐varying covariate at a given time t was $Z_{i1}(t)=\tau_{i}\sin(2t)$, where $\tau_{i}∼N(1,1)$. This condition had true parameter values of $\beta_{1}=-0.5$ and $\gamma_{1}=1$.
  Note that the TM approach assumes that measurements of the time‐varying covariate(s) and the event status of the individual are both taken at a series of known observation times. To facilitate this, for each of these conditions, we simulated a number of observation times $n_{i}$ for individual i from a Poisson distribution, and the intervals between the observation times were found by taking $n_{i}$ draws from a $\text{Unif}[\tau_{1},\tau_{2}]$ distribution. At each of these assessment times, the value of the time‐varying covariates were recorded, as was the event status of the individual i. Simulation results from Conditions 1 & 3 are reported in this paper and from Condition 2 are included in the Supplementary Material.

### 4.2 Results

Results for Study 1 associated with the Gompertz baseline hazard function are reported in Tables 1 and 2, and results of this study associated with the exponential baseline hazard function are available in the Supplementary Material.

**TABLE 1:** Study 1 (right censoring): Regression parameters of MPL and PL methods with Gompertz baseline for n=200 and n=1000

		n=200						n=1000					
		$\pi^{E}=0.7$			$\pi^{E}=0.3$			$\pi^{E}=0.7$			$\pi^{E}=0.3$		
		Bias	SE	CP	Bias	SE	CP	Bias	SE	CP	Bias	SE	CP
$\beta_{1}$	MPL	0.0512	0.1765	0.94	0.1252	0.2781	0.90	0.0014	0.0785	0.96	0.0422	0.1203	0.92
			(0.1773)			(0.3150)			(0.1008)			(0.1327)	
	PL	−0.2496	0.1491	0.60	−0.6875	0.1444	0.00	−0.2621	0.0723	0.06	−0.6961	0.0636	0.00
			(0.1587)			(0.1477)			(0.0718)			(0.0700)	
$\beta_{2}$	MPL	0.0425	0.2907	0.93	0.1132	0.4325	0.92	0.0089	0.1300	0.92	0.0206	0.1910	0.94
			(0.3117)			(0.4774)			(0.1438)			(0.1995)	
	PL	0.1084	0.2521	0.91	0.3437	0.2508	0.71	0.1286	0.1158	0.79	0.3182	0.1105	0.20
			(0.2755)			(0.2517)			(0.1237)			(0.1121)	
$\gamma_{1}$	MPL	−0.0247	0.3676	0.93	0.0056	0.4597	0.93	0.0065	0.1936	0.92	−0.0094	0.2279	0.92
			(0.3921)			(0.4904)			(0.2259)			(0.2711)	
	PL	−0.0195	0.5160	0.96	−0.0924	4.0216	0.97	0.0058	0.2170	0.95	−0.0083	0.2499	0.94
			(0.5438)			(0.9168)			(0.2206)			(0.2678)	

Abbreviations: CP, coverage probability; SE, standard error.

**TABLE 2:** Study 1 (right censoring): Baseline survival function results with Gompertz baseline for n=200 and n=1000

		n=200						n=1000					
		$\pi^{E}=0.7$			$\pi^{E}=0.3$			$\pi^{E}=0.7$			$\pi^{E}=0.3$		
		Bias	SE	CP	Bias	SE	CP	Bias	SE	CP	Bias	SE	CP
$t_{1}$	MPL	0.0079	0.0869	0.97	0.0133	0.0330	0.95	‐0.0030	0.0587	0.99	0.0046	0.0163	0.96
	PL	0.0164	0.0952	0.97	−0.0231	0.0226	0.82	0.0452	0.0182	0.31	−0.0238	0.0111	0.43
$t_{2}$	MPL	0.0156	0.1299	0.97	0.0207	0.0647	0.96	0.0019	0.0737	0.96	0.0070	0.0308	0.94
	PL	0.0322	0.1455	0.96	−0.0361	0.0368	0.85	0.0636	0.0351	0.54	−0.0403	0.0201	0.51
$t_{3}$	MPL	0.0254	0.1177	0.96	0.0274	0.1002	0.95	0.0059	0.0642	0.94	0.0067	0.0517	0.96
	PL	0.0524	0.1315	0.94	−0.5185	0.1243	0.03	0.0611	0.0366	0.60	−0.4610	0.0737	0.00

*Note*: $t_{1}=25^{th}$ percentile of baseline distribution; $t_{2}=50^{th}$ percentile of baseline distribution; $t_{3}=75^{th}$ percentile of baseline distribution.

Table 1 gives the bias, asymptotic and (Monte Carlo) (in brackets) standard error estimates, and the asymptotic coverage probabilities for the regression parameters from Study 1. The MPL estimates are generally less biased compared to the PL estimates, particularly for the smaller sample size. Both the MPL and PL methods have reasonable agreement between the asymptotic and Monte Carlo standard error estimates. However, the MPL method provides superior coverage probabilities, especially when censoring is higher. These results are in agreement with those reported in Reference 7.

Table 2 gives the bias, Monte Carlo standard errors and the Monte Carlo coverage probabilities for baseline survival function values at three selected time points: 25% (denoted by $t_{1}$), 50% ($t_{2}$) and 75% ($t_{3}$) percentiles of the baseline distribution (ie, the distribution associated with the baseline hazard) across the simulated datasets. Note that for this part of the simulation results asymptotic standard error estimates of the baseline survival function are not included because they are unavailable for the PL method, although they are available from the MPL method. Clearly, the bias in the MPL baseline survival function estimates are consistently smaller than the bias from the PL method. Remarkably, the Monte Carlo coverage probabilities for the MPL estimates are all close to the nominal value of 0.95 while these coverage probabilities can be extremely poor for the PL estimates, especially when censoring is higher.

**FIGURE 1:**
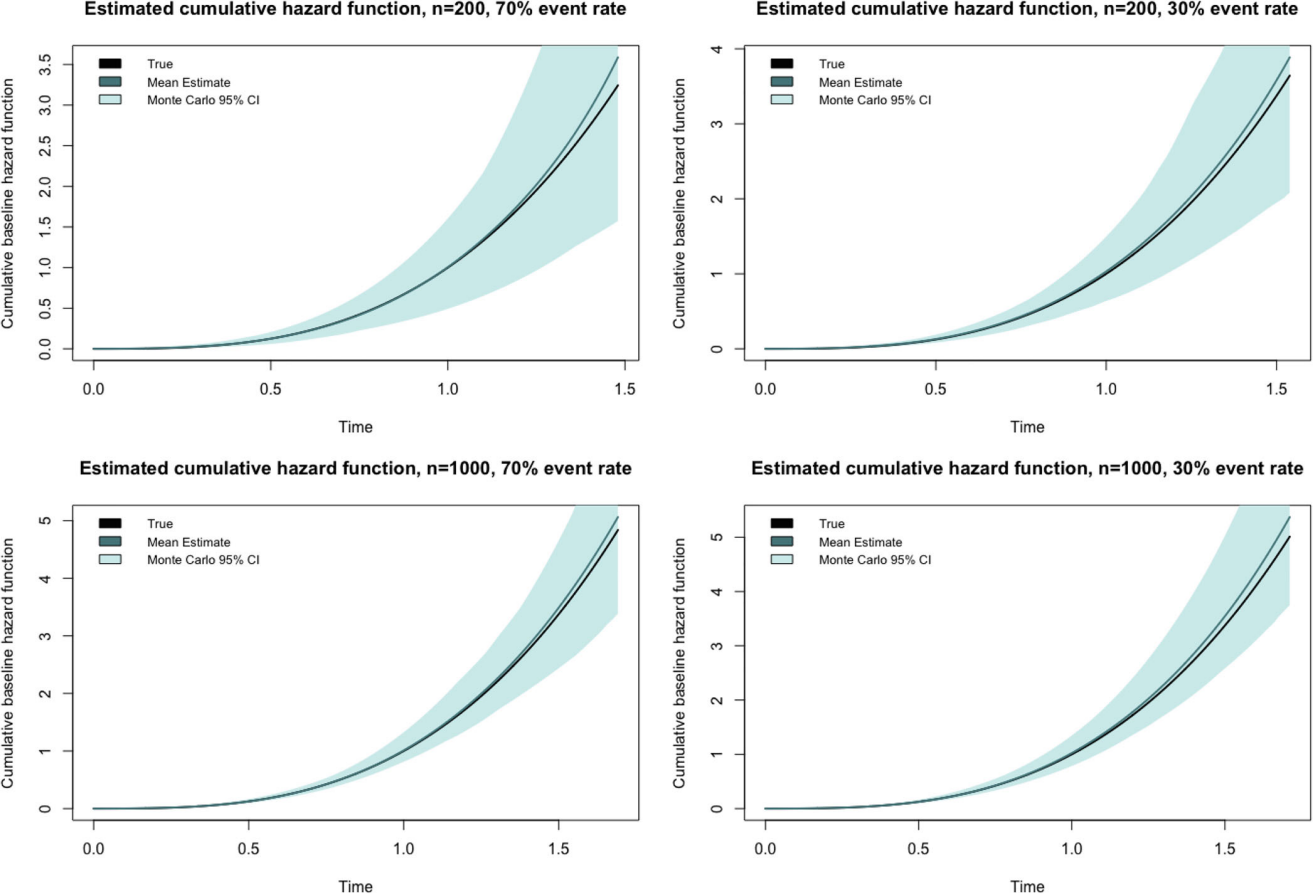
Study 2, Scenario 1 (partly‐interval censoring): True vs estimated baseline cumulative hazard function for Weibull baseline hazard function

Results for Study 2 Scenario 1 for the Weibull baseline hazard function are provided in Tables 3 and 4, whilst the results for the log‐logistic baseline hazard are available in the Supplementary Material. Table 3 shows the bias, asymptotic and (Monte Carlo) standard errors and asymptotic coverage probabilities for the time‐fixed regression parameters (ie, $\beta_{1}$ and $\beta_{2}$) and the time‐varying coefficient (ie, $\gamma_{1}$). The results in Table 3 demonstrate that the MPL method achieves small biases for $\beta_{1}$ and $\beta_{2}$ even when the sample size is small and censoring proportion is large. In general, compared to the results of $\beta_{1}$ and $\beta_{2}$, the MPL estimate of $\gamma_{1}$ can produce higher biases with less agreement between asymptotic and Monte Carlo standard errors when the sample size is small. It also gave slightly poorer coverage probabilities than the time‐fixed covariates coefficients. Table 4 reports the results of baseline survival function corresponding to $t_{1}$ (25th percentile), $t_{2}$ (50th percentile) and $t_{3}$ (75th percentile) of the baseline distribution. Particularly, biases and standard errors (both asymptotic and (Monte Carlo)) for the estimated baseline survival function values at these three points are included. The results show that the biases in the baseline survival estimates are small, even when the sample size is small or when the censoring proportion is large. The coverage probabilities tend to decrease when percentiles increase, likely to be a consequence of more right censoring times at the large percentiles. In Figure 1, we plotted the mean estimated cumulative baseline hazard function and its Monte Carlo 95% confidence interval against the true cumulative baseline hazard function. These plots show that across both sample sizes and event rates, the true cumulative baseline hazard function lies within the Monte Carlo 95% confidence interval of the estimate. In the Supplementary Material Table 7, results from the small sub‐study performed on samples where only a small proportion of the sample ever have a value of $Z_{1}=1$ show that the bias in the regression parameter $\gamma_{1}$ estimate is not affected, but that this does sometimes produce low asymptotic variance estimates, and poorer coverage probabilities as a result, especially when the sample size is small.

**TABLE 3:** Study 2, Scenario 1 (partly‐interval censoring): Regression parameters of MPL method with Weibull baseline hazard function for n=200 and n=1000

	n=200						n=1000					
	$\pi^{E}=0.7$			$\pi^{E}=0.3$			$\pi^{E}=0.7$			$\pi^{E}=0.3$		
	Bias	SE	CP	Bias	SE	CP	Bias	SE	CP	Bias	SE	CP
$\beta_{1}$	−0.0048	0.1690	0.95	−0.0147	0.2174	0.96	0.0018	0.0790	0.95	−0.0084	0.0983	0.95
		(0.1706)			(0.2003)			(0.0867)			(0.0993)	
$\beta_{2}$	−0.0415	0.2838	0.94	0.0238	0.3672	0.96	0.0057	0.1250	0.92	0.0273	0.1583	0.92
		(0.2861)			(0.3731)			(0.1405)			(0.1708)	
$\gamma_{1}$	0.0934	0.1996	0.92	0.1276	0.2650	0.87	0.0141	0.1073	0.92	0.0076	0.1436	0.88
		(0.2189)			(0.3143)			(0.1151)			(0.1814)	

**TABLE 4:** Study 2, Scenario 1 (partly‐interval censoring): Baseline survival function results from MPL method with Weibull baseline hazard function for n=200 and n=1000

	n=200						n=1000					
	$\pi^{E}=0.7$			$\pi^{E}=0.3$			$\pi^{E}=0.7$			$\pi^{E}=0.3$		
	Bias	SE	CP	Bias	SE	CP	Bias	SE	CP	Bias	SE	CP
$t_{1}$	−0.0097	0.0218	0.94	−0.0061	0.0225	0.89	0.0008	0.0138	0.94	0.0015	0.0140	0.91
		(0.0323)			(0.0323)			(0.0274)			(0.0316)	
$t_{2}$	0.0050	0.0749	0.93	0.0174	0.0884	0.93	−0.0015	0.0308	0.91	0.0056	0.0440	0.86
		(0.1091)			(0.1232)			(0.0701)			(0.0990)	
$t_{3}$	0.0179	0.0465	0.91	0.0358	0.0727	0.90	0.0014	0.0108	0.90	0.0048	0.0229	0.85
		(0.0669)			(0.1131)			(0.0195)			(0.0424)	

*Note*: $t_{1}=25^{th}$ percentile of baseline distribution; $t_{2}=50^{th}$ percentile of baseline distribution; $t_{3}=75^{th}$ percentile of baseline distribution.

Tables 5 and 6 show the results for Study 2, Scenario 2 under Conditions 1 & 3, and these results are used to compare the performance of the MPL method with the TM approach of Reference 15. Table 5 shows that the bias in the MPL estimates is similar or less than the TM method, and is especially smaller for the estimates of the time‐varying covariate coefficient $\gamma_{1}$. Both methods show fair agreement between the asymptotic and Monte Carlo standard error estimates, particularly in the larger sample. The 95% coverage probabilities for the $\gamma_{1}$ parameter are somewhat low for both methods, although are particularly low for the TM based estimate of $\gamma_{1}$ when the time‐varying covariate is continuous. Table 6 shows that the MPL estimation of the baseline cumulative hazard function has consistently lower bias than the step‐wise estimation used by Reference 15. Additionally, the Monte Carlo coverage probabilities for the MPL method are reasonable, but are noticeably low for the TM estimates. When the time‐varying covariate is a continuous function of time and the sample size is larger, the Monte Carlo coverage probabilities for the baseline cumulative hazard function are high for both methods. Results for Condition 2, considering two time‐varying covariates, are available in the Supplementary Material. These results indicate that the addition of a second time‐varying covariate may increase the bias in all estimates for both the MPL and TM methods compared to scenarios with one time‐varying covariate, although the bias in the estimates is still noticeably smaller for the MPL method than the TM method, especially for the time‐varying covariate coefficients.

**TABLE 5:** Study 2, Scenario 2 (interval censoring): Regression parameters of MPL and transformation model (TM) method for Weibull baseline hazard function, for n=200 and n=1000

		n=200			n=1000		
		Bias	SE	CP	Bias	SE	CP
Discrete time‐varying covariate							
$\beta_{1}$	MPL	0.024	0.320	0.94	0.015	0.139	0.95
			(0.340)			(0.133)	
	TM	−0.068	0.341	0.94	0.002	0.141	0.93
			(0.360)			(0.150)	
$\beta_{2}$	MPL	0.075	0.468	0.98	0.034	0.213	0.98
			(0.383)			(0.222)	
	TM	0.078	0.539	0.98	0.050	0.229	0.97
			(0.462)			(0.235)	
$\gamma_{1}$	MPL	0.054	0.370	0.84	‐0.010	0.176	0.83
			(0.558)			(0.242)	
	TM	−0.161	0.495	0.91	−0.257	0.229	0.78
			(0.67)			(0.264)	
Continuous time‐varying covariate							
$\beta_{1}$	MPL	−0.012	0.192	0.95	−0.037	0.082	0.81
			(0.211)			(0.109)	
	TM	0.037	0.266	0.99	0.019	0.085	0.96
			(0.214)			(0.079)	
$\gamma_{1}$	MPL	0.062	0.142	0.90	0.048	0.061	0.89
			(0.160)			(0.055)	
	TM	−0.311	0.192	0.71	−0.155	0.058	0.20
			(0.194)			(0.052)	

**TABLE 6:** Study 2, Scenario 2 (interval censoring): Cumulative hazard function results from MPL and transformation model (TM) method for Weibull baseline hazard, for n=200 and n=1000

		n=200			n=1000		
		Bias	SE	CP	Bias	SE	CP
Discrete time‐varying covariate							
$t_{1}$	MPL	−0.007	0.028	0.97	−0.002	0.004	0.93
	TM	−0.027	0.031	0.89	0.018	0.017	0.57
$t_{2}$	MPL	−0.022	0.069	0.97	0.006	0.036	0.95
	TM	−0.070	0.051	0.69	0.033	0.032	0.48
$t_{3}$	MPL	−0.040	0.155	0.97	0.038	0.066	0.93
	TM	−0.143	0.113	0.70	−0.124	0.068	0.53
Continuous time‐varying covariate							
$t_{1}$	MPL	0.008	0.011	0.94	−0.011	0.158	1.00
	TM	0.009	0.022	0.92	0.013	0.022	0.97
$t_{2}$	MPL	0.007	0.058	0.98	−0.009	0.131	1.00
	TM	0.038	0.044	0.83	0.037	0.064	0.99
$t_{3}$	MPL	−0.005	0.108	0.99	−0.004	0.189	1.00
	TM	0.099	0.087	0.81	0.075	0.170	1.00

*Note*: $t_{1}=25^{th}$ percentile of baseline distribution; $t_{2}=50^{th}$ percentile of baseline distribution; $t_{3}=75^{th}$ percentile of baseline distribution.

## 5 APPLICATIONS

### 5.1 Bangkok HIV study data

In this section, we illustrate the use of the proposed method by applying it to a real data set containing discrete time‐varying covariates. The data set used in this section is from a cohort study carried out by the Bangkok Metropolitan Administration, which followed 1209 injecting drug users over the period of 1995 to 1998 until HIV positive. This data has previously been analysed in Reference 15. Participants were included in the study if they were HIV negative at the time of enrollment, and were then repeatedly tested for HIV at approximately four month intervals. This means the event times were all either interval‐ or right‐censored. At baseline, four time‐fixed covariates were recorded: age at enrollment (in years), sex (male or female), whether the patient had a history of injecting drug use (yes or no), and whether the patient had a history of sharing needles. At each follow up, in addition to HIV status, a discrete time‐varying covariate was also recorded: whether an individual had been in jail since the previous follow up (yes or no). A total of 133 patients (11%) experienced the event of interest during the follow‐up, and the remaining patients were right‐censored, which means this is a heavily right‐censored sample.

We used our proposed method to investigate which of these variables were significant predictors of HIV risk. In Table 7, we present the parameter estimate, asymptotic standard error estimate, and p‐value of each of the covariates from our proposed MPL method, alongside the estimates fitted using the TM approach of Reference 15. The magnitude and direction of the parameter estimates for each covariate are similar between the MPL and TM methods: each additional year of age reduces the risk of developing HIV, and having been in jail since the last follow‐up increases the risk of developing HIV.

**TABLE 7:** Bangkok HIV study survival analysis results

	MPL Cox model			Transformation Cox model		
Variable	Estimate	SE	p‐value	Estimate	SE	p‐value
Age	−0.020	0.008	0.002	−0.029	0.012	0.016
Gender: Male	−0.087	0.194	0.653	−0.121	0.196	0.536
Injecting history: Yes	−0.024	0.112	0.834	0.031	0.110	0.774
Needle sharing history: Yes	−0.084	0.233	0.720	−0.106	0.195	0.585
Jail: Yes	1.282	0.211	<0.001	1.226	0.181	<0.001

**FIGURE 2:**
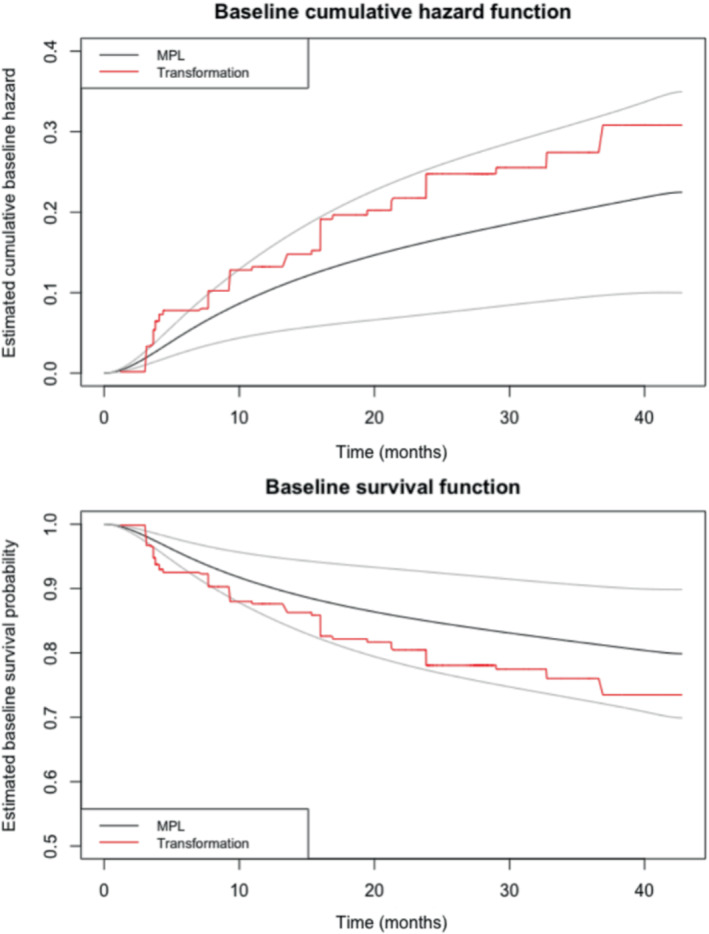
Estimated baseline cumulative hazard and baseline survival functions, with point‐wise 95% confidence intervals for MPL estimates, for Bangkok HIV study

To compare the smooth estimate of the baseline distribution quantities provided by our MPL method with the step‐wise estimation of the TM method, we compared their estimates of the baseline survival and cumulative baseline hazard functions. Figure 2 shows the estimates of the cumulative baseline hazard and baseline survival functions along with their point‐wise 95% confidence intervals from the MPL model, as well as the step‐wise estimate from the TM method. Clearly, the MPL baseline cumulative hazard function and survival function estimates are smooth. The estimates of these functions from the TM method is somewhat different from that of MPL, but they both are still within the MPL point‐wise confidence intervals, so the differences may not be significant.

**FIGURE 3:**
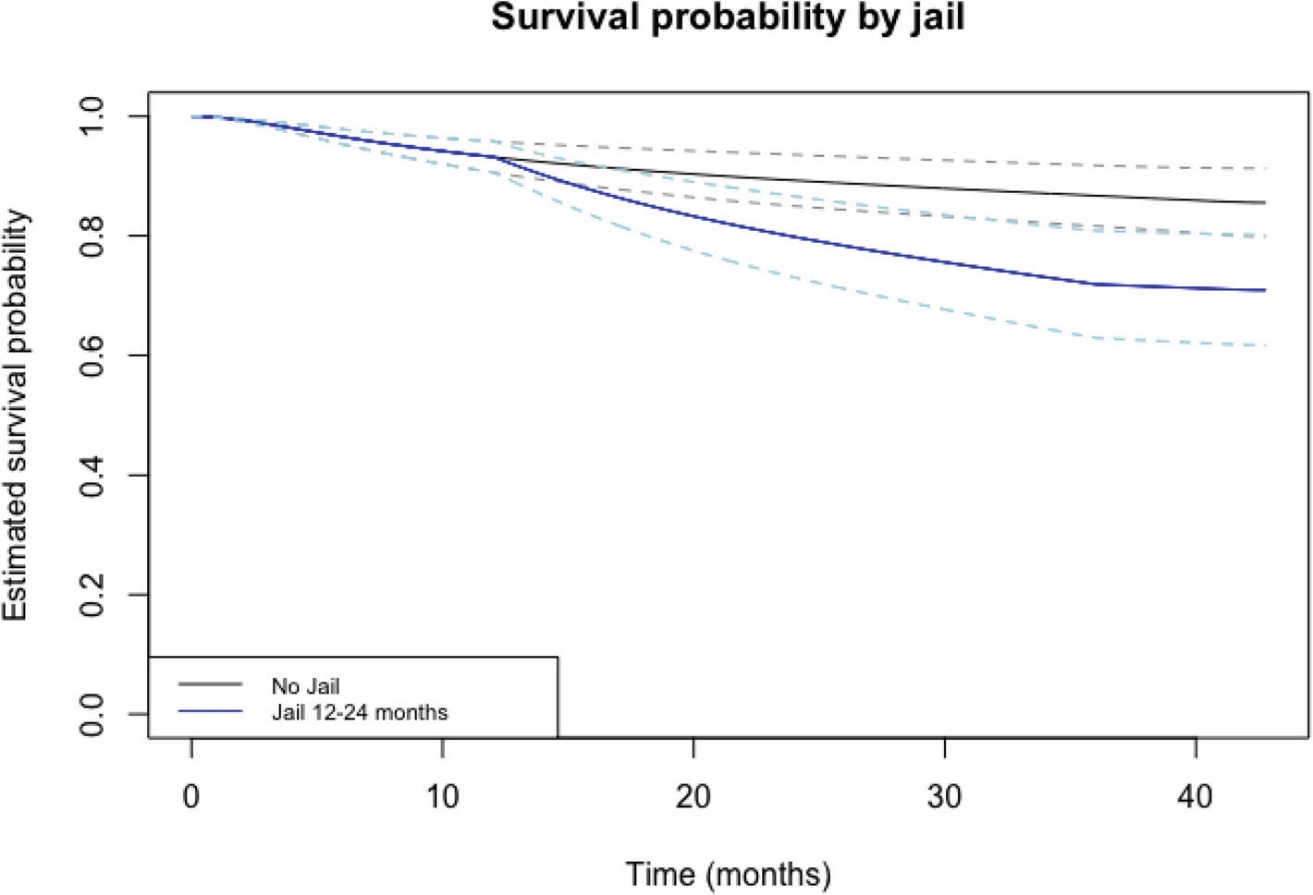
Comparative estimated survival functions with point‐wise 95% confidence intervals for Bangkok HIV study

A key strength of the spline estimation of the baseline survival quantities provided by the MPL method is the ability to make predictions of survival probabilities at a given time t. To demonstrate this, we display in Figure 3 the predicted survival functions for individuals under two different groups: the first group is of individuals who were never in jail during the follow‐up period, and second group is of individuals who were in jail between month five and month ten of the follow‐up period. In both of these two groups, we set age to 18 years, and set the remaining baseline (time‐fixed) covariates to zero. We calculated point‐wise 95% confidence intervals for these predicted survival curves using the delta method and plotted these alongside the estimated survival curves. Clearly, time spent in jail decreases the probability that an individual will be HIV negative at a given time. For example, at the end of the follow‐up (40 months), the individual who had never spent time in jail had a probability of being HIV negative of 85.56% (95% CI: 79.82%, 91.29%). For the individual who had spent time in jail, this probability is 70.92% (95% CI: 61.69%, 80.15%).

### 5.2 Breast cancer recurrence data

We also applied our proposed method to another data set containing interval‐censored cancer recurrence times and discrete time‐varying covariates. This data set was obtained from The Cancer Imaging Archive and contains retrospective data on 902 breast cancer patients at Duke Hospital, USA, collected from 2000 to 2014. The full data set contains a large number of variables and is described in more detail in Reference 30. For the purposes of illustrating our method, we considered only a subset of the demographic and clinical factors recorded. These included four baseline covariates: age at diagnosis (<35 years; ≥35 years), the extent of spread to the lymph nodes at diagnosis (Stage N0 or N1; Stage N2 or N3), whether breast cancer sub‐type was triple negative (yes; no), and the size/location of the tumour at diagnosis (Stage T0, T1, T2, or T3; Stage T4). Additionally, two time‐varying covariates were included, indicating whether a patient had undergone one of the two possible surgical interventions: breast conservation surgery (BCS) (yes; no), or mastectomy (yes; no). The vast majority (98%) of patients underwent surgery of some type, with 52% undergoing breast conservation surgery and 46% undergoing mastectomy; note that no patients in the data set underwent both types of surgery. The event of interest was time to breast cancer recurrence of any kind. For patients who had experienced breast cancer recurrence during the follow‐up period, the last recurrence‐free assessment time and the time at which recurrence was diagnosed were both recorded, and these individuals were interval‐censored. All patients who did not experience recurrence during the follow‐up period were considered right‐censored. Approximately 10% of patients in the data set experienced a recurrence during the follow‐up, again a large proportion of right censoring.

We again analyzed this data using both our proposed MPL method and the TM method. In Table 8, we present the parameter estimates, asymptotic standard errors and p‐values obtained from both methods. They display similar regression coefficients for time‐fixed covariates, but their time‐varying covariates coefficients are noticeably different. According to both methods, having the triple negative sub‐type, having greater lymph node involvement, and being diagnosed with Stage T4 cancer rather than an earlier stage all significantly increase the risk of breast cancer recurrence. Additionally, both methods indicate that being older than 35 years at diagnosis significantly decreases the risk of recurrence. The MPL model suggests that both types of surgery significantly decrease the risk of recurrence, with mastectomy, the more aggressive of the two surgery types, having the larger effect on risk. However, neither of the surgery types are significant predictors of risk in the TM method. Additionally, it is notable that the TM method has produced relatively large standard error estimates for these two time‐varying covariates.

**TABLE 8:** Breast cancer recurrence survival analysis results

	MPL Cox model			Transformation Cox model		
Variable	Estimate	SE	p‐value	Estimate	SE	p‐value
Age: ≥35	−0.485	0.258	<0.001	−0.675	0.314	0.031
Triple negative: Yes	0.780	0.254	<0.001	0.714	0.229	0.002
Lymph involvement: Stage N2 or N3	1.260	0.238	<0.001	1.192	0.234	<0.001
Tumour size/Location: Stage 4	1.356	0.439	<0.001	1.191	0.362	0.001
BCS: Yes	−1.951	0.034	<0.001	−0.616	1.092	0.573
Mastectomy: Yes	−2.077	0.063	<0.001	−0.219	1.091	0.841

**FIGURE 4:**
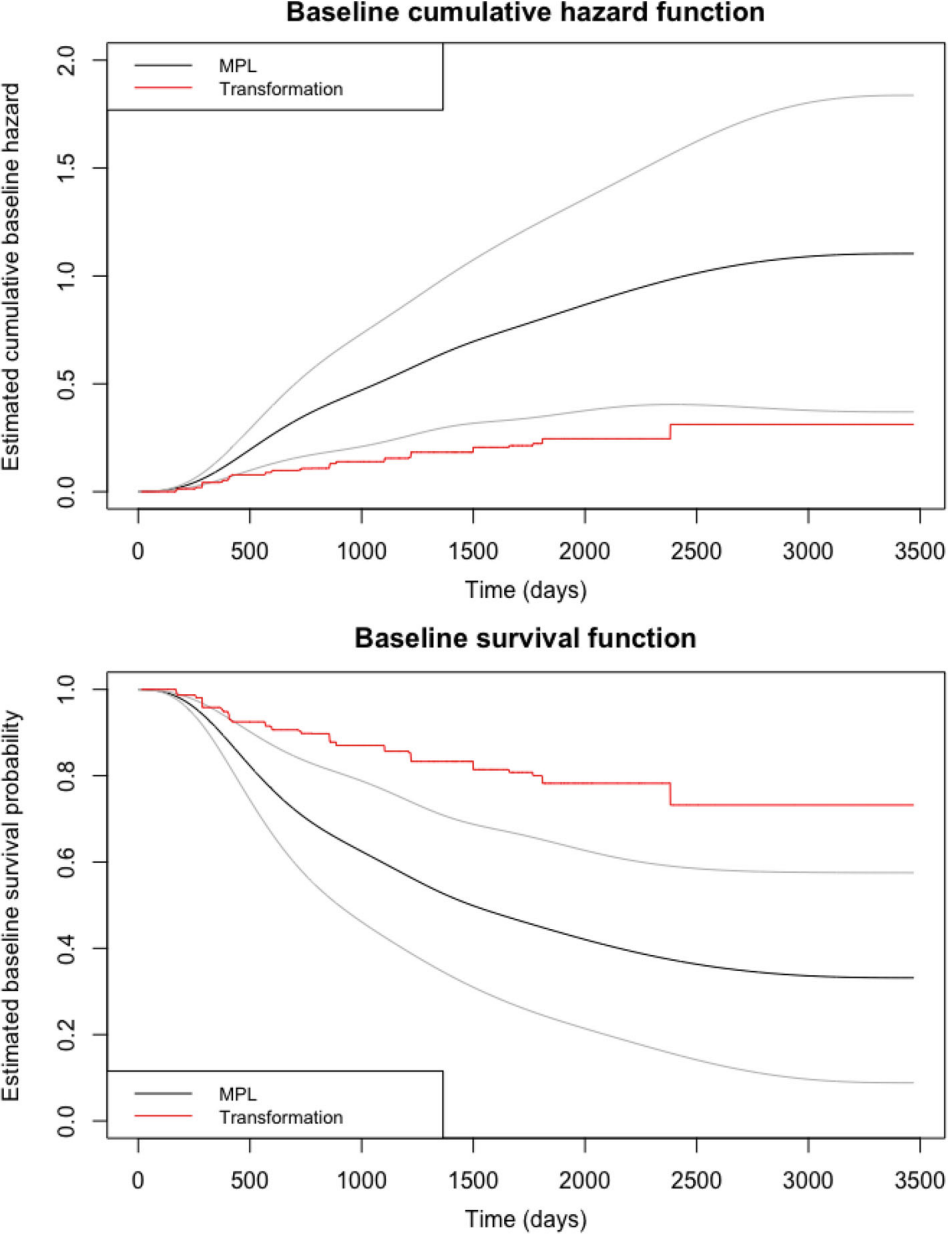
Estimated baseline cumulative hazard and baseline survival functions, with point‐wise 95% confidence intervals for MPL estimates, for the breast cancer recurrence study

We also estimated the baseline cumulative hazard and baseline survival functions using our MPL method and plotted in them in Figure 4 alongside their point‐wise 95% confidence intervals, and the step‐wise estimates of these functions from the TM estimation. From the MPL estimates, it appears that the baseline risk of recurrence is small for a short period at the beginning of the follow‐up period for approximately the first 100 days, increases sharply for a short time after that until approximately 600 days, increases less rapidly until approximately 2750 days, and then begins to level off. It is clear from the plots that there is a large discrepancy between the MPL and the TM method, with the TM estimates laying outside the MPL point‐wise confidence intervals. This discrepancy is a reflection of the different regression parameter estimates for both surgery types between these two methods, and subsequent differences in the risk of recurrence in the “baseline" group.

**FIGURE 5:**
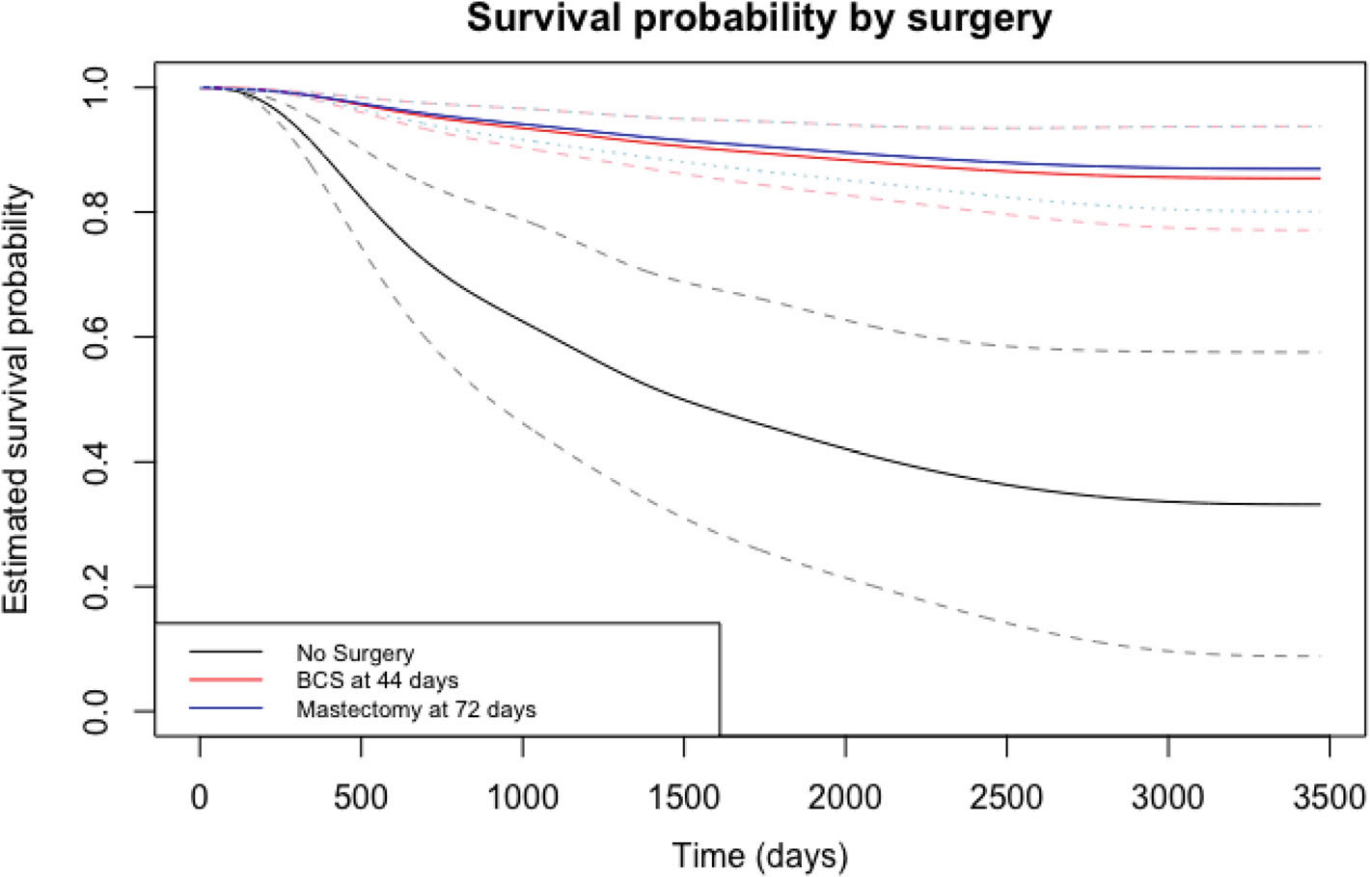
Comparative estimated survival functions with point‐wise 95% confidence intervals for breast cancer recurrence study

We also use our MPL method for predictive survival analysis, with plots displayed in Figure 5. Specifically, for this data, we compared the survival probabilities for an individual who never had either surgery type, an individual who had breast conservation surgery at the median surgery time for this type (44 days), and an individual who had a mastectomy at the median surgery time for this type (72 days), where all baseline (time‐fixed) covariates were set to zero. Figure 4 shows the predicted survival functions for these individuals along with 95% confidence intervals. It is clear from the plot that either surgery type greatly increases the probability of recurrence‐free survival across the follow‐up, and that these differences in survival compared to those who had no surgery appear significant at the 5% level. Mastectomy offers a small increase in probability of recurrence‐free survival compared to breast‐conservation surgery, but the difference between mastectomy and breast conservation surgery does not appear to be significant.

## 6 DISCUSSION AND CONCLUDING REMARKS

In this paper we have presented a novel method for fitting a Cox model to partly‐interval censored data with time‐varying covariates. Unlike the transformation model method, this MPL approach makes no assumption about the relationship between the monitoring of the time‐varying covariates and the event of interest, and is thus more flexible and applicable in a wider variety of study designs. Additionally, the penalised likelihood estimation of this model produces a smooth baseline hazard estimate and allows for asymptotic variance estimates to be obtained for both regression parameters and survival quantities. The results of our simulation studies demonstrate that our proposed method performs well when applied to both right‐ and partly interval‐censored data. We have provided two examples of how our method can be applied to real data, demonstrating the utility of our method especially for prediction.

While our simulation study results show that our method works well for both discrete and continuous time‐varying covariates, an obvious limitation of our proposed method is that it does not account for measurement error in the observation of these time‐varying covariates. Joint modelling of survival and longitudinal data (see References 31, 32, 33 among many others) is a popular approach for accounting for potential measurement error in time‐varying covariates used in Cox models. In future work we intend to expand the model and MPL estimation approach proposed here to a joint modeling framework so as to incorporate continuous longitudinal covariates with possible measurement error in their observation. Joint modelling approaches for partly‐interval censored survival data have been proposed,^34^, ^35^ but we note that these existing methods do not provide asymptotic variance estimates for regression parameters or survival quantities, nor a smooth estimate of the baseline hazard function.

## Supporting information




**Appendix S1**: Supporting Information.

## Data Availability

The R code used for the simulation studies and data analysis in this paper is publicly available at github.com/annabelwebb/tvc_mpl. Both datasets analysed in this paper are also publicly available. The HIV data set is available in the Supplementary Material of Reference 15, as is the R package required to fit the transformation model considered in the paper. The breast cancer recurrence data is available from doi.org/10.7937/TCIA.e3sv‐re93.
