# Supplementary material for: Cox models with time‐varying covariates and partly‐interval censoring–A maximum penalised likelihood approach
Source: Stat Med. 2022 Dec 30;42(6):815–33. doi: 10.1002/sim.9645 (PMC10107645; doi:10.1002/sim.9645)
Supplement: Supplementary file 1 — Appendix S1: Supporting Information. [file SIM-42-815-s001.pdf]

Supplementary Material for: Cox models with time-varying covariates and partly-interval censoring – a maximum penalised likelihood approach

Annabel Webb[1] and Jun Ma[2]

[1, 2]Department of Mathematics and Statistics, Macquarie University, Australia

1 Score vector and Hessian matrix elements

Here we provide further details of the necessary elements for computing $\boldsymbol{\beta}^{(k+1)}$, $\boldsymbol{\gamma}^{(k+1)}$ and $\boldsymbol{\theta}^{(k+1)}$ in our Newton-MI algorithm, outlined in Section 3 of the main paper.

Firstly we detail the values of $\mathbf{c}$ and $\mathbf{a}$ which form the diagonals of the $n \times n$ matrices $\mathbf{C}$ and $\mathbf{A}$, needed to update the parameter vector $\boldsymbol{\beta}$. We have

$$\mathbf{c} = \mathbf{H} * \boldsymbol{\delta}^* + \mathbf{H}_I * \boldsymbol{\delta}_I^*,$$

where $\mathbf{H}$ and $\mathbf{H}_I$ are vectors whose elements are $H(y_i)$ and $\delta_i^I H(t_i^L)$ respectively, $\boldsymbol{\delta}^*$ and $\boldsymbol{\delta}_I^*$ are vectors whose elements are $\delta_i^* = \delta_i + \delta_i^R - \delta_i^L S(y_i)/(1 - S(y_i)) - \delta_i^I S(t_i^R)/(S(t_i^L) - S(t_i^R))$ and $\delta_{Ii}^* = \delta_i^I S(t_i^L)/(S(t_i^L) - S(t_i^R))$ respectively. Here, '$*$' denotes element-wise multiplication throughout this paper. For $\mathbf{a}$ we have

$$a_i = c_i + \delta_i^L \frac{H^2(y_i)S(y_i)}{(1 - S(y_i))^2} + \delta_i^I \frac{(H(t_i^L) - H(t_i^R))^2 S(t_i^L)S(t_i^R)}{(S(t_i^L) - S(t_i^R))^2},$$

where c_i is the ith element of vector $\mathbf{c}$. Clearly, if a sample only has event times and right censoring times (i.e. $\delta_i^L = \delta_i^I = 0$) then the matrices $\mathbf{A}$ and $\mathbf{C}$ are identical.

Next we detail the value of vector $\boldsymbol{\epsilon}^E$ and the block diagonal matrices $\mathbf{B}$ and $\mathbf{E}$, required for updating the estimate of the parameter vector $\boldsymbol{\gamma}$. This computation requires us to establish some additional notation. Where there are interval censored observations in a data set, it is necessary to construct a long format data frame which contains rows corresponding to interval censoring observations truncated at t_i^L. Consider for individual i a vector of length $n_i + 1$ that gives all the values of t_{ia} for $a = 1, ..., n_i$ as well as y_i, given as $[t_{i1}, t_{i2}, ..., t_{in_i}, y_i]^T$. Then say that the number of $t_{ia} < t_i^L$ is equal to c_i. Let τ_{c_i} be a vector of length c_i with values $[t_{i1}, ..., t_{ic_i}]^T$. The truncated list of times can then be given as a vector of length $n_i + 1$, with values $\tau_i = [\tau_{c_i}^T, t_i^L, \mathbf{0}_{(n_i - c_i)}^T]^T$ where $\mathbf{0}_{(n_i - c_i)}$ is a vector of zeroes of length $n_i - c_i$. Note that if individual i is not interval censored, then $c_i = 0$ and $\tau_i = \mathbf{0}_{n_i}$. It is also convenient to establish notation for what could be considered the *long format* versions of the event and censoring type

indicators for individual i, $(\delta_i, \delta_i^R, \delta_i^L, \delta_i^I)$. These can be represented as $(\boldsymbol{\epsilon}_i, \boldsymbol{\epsilon}_i^R, \boldsymbol{\epsilon}_i^L, \boldsymbol{\epsilon}_i^I)$. $\boldsymbol{\epsilon}_i$ is an n_i-vector equal to $\delta_i \mathbf{1}_{n_i}$. We can then denote the N-vector $\boldsymbol{\epsilon} = [\boldsymbol{\epsilon}_1^T, ..., \boldsymbol{\epsilon}_n^T]^T$ for the whole sample. Similarly, we can obtain $\boldsymbol{\epsilon}^R, \boldsymbol{\epsilon}^L, \boldsymbol{\epsilon}^I$ for right-, left- and interval-censoring respectively.

Now, we can express the vector $\boldsymbol{\epsilon}^E = \boldsymbol{\zeta} * \boldsymbol{\epsilon}$ with $\boldsymbol{\zeta}$ being an N-vector with 1's at indices $n_1, (n_1+n_2), \ldots, N$ and 0's elsewhere (i.e. 1's at indices corresponding to the last record for each i) and $\boldsymbol{\epsilon}$ as just established. Additionally, we have $\mathbf{E}$, an N-vector given by $\mathbf{E} = (\mathbf{E}_1^\top, ..., \mathbf{E}_n^\top)$. The vector $\mathbf{E}_i$ has the dimensions $n_1 \times 1$ and is given by $\mathbf{E}_i = \mathbf{H}_i^* * \boldsymbol{\epsilon}_i^* + \mathbf{H}_{Ii}^* * \boldsymbol{\epsilon}_{Ii}^*$. $\mathbf{H}_i^*$ and $\mathbf{H}_{Ii}^*$ are both n_i-vectors with elements $e^{x_i^T \boldsymbol{\beta} + z_{ia}^T \boldsymbol{\gamma}}[H_0(t_{i,a+1}) - H_0(t_{ia})]$ and $e^{x_i^T \boldsymbol{\beta} + z_{ia}^T \boldsymbol{\gamma}}[H_0(\tau_{i,a+1}) - H_0(\tau_{ia})]$ respectively, and $\boldsymbol{\epsilon}_i^*$ and $\boldsymbol{\epsilon}_{Ii}^*$ are also both n_i vectors with $\boldsymbol{\epsilon}_i^* = \boldsymbol{\epsilon}_i + \boldsymbol{\epsilon}_i^R - S(y_i)/(1 - S(y_i))\boldsymbol{\epsilon}_i^L - S(t_i^R)/(S(t_i^L) - S(t_i^R)\boldsymbol{\epsilon}_i^I)$ and $\boldsymbol{\epsilon}_{Ii}^* = S(t_i^L)/(S(t_i^L) - S(t_i^R))\boldsymbol{\epsilon}_i^I$. Recall that $a = 0, \ldots, n_i - 1$ and that τ_{ia} are the changing points in $\mathbf{z}_i(t)$ truncated at t_i^L as defined above. Then, the matrix $\mathbf{B}$ is an $N \times N$ block diagonal matrix given by $\mathbf{B} = \text{diag}(\mathbf{B}_1, ..., \mathbf{B}_n)$, and each $\mathbf{B}_i$ is a diagonal matrix, with its diagonal given by the n_i-vector

$$\mathbf{E}_i + \frac{S(y_i)}{(1 - S(y_i))^2}\boldsymbol{\epsilon}_i^L * \mathbf{H}_i^* * \mathbf{H}_i^* - \frac{S(t_i^L)S(t_i^R)}{(S(t_i^L) - S(t_i^R))^2}\boldsymbol{\epsilon}_i^I * (\mathbf{H}_{Ii}^* - \mathbf{H}_i^*) * (\mathbf{H}_{Ii}^* - \mathbf{H}_i^*).$$

Finally, we detail the necessary computations for updating the parameter vector $\boldsymbol{\theta}$. Let $\mathbf{p}$, $\mathbf{p}^*$ and $\mathbf{p}_I^*$ all be $n \times m$ matrices. The $(i, u)^{th}$ elements of these matrices are, respectively, $\psi_u(y_i)$, $\Psi_{ui}^*(y_i)$ and $\delta_i^I \Psi_{ui}(t_i^L)$. Let $\mathbf{e}$ be an n-vector for the $e^{x_i^\top \boldsymbol{\beta}}$'s, and let $\mathbf{f}$ be an $n \times n$ diagonal matrix with $(i, i)^{th}$ element $h_0(y_i)$. Additionally, let $\mathbf{J}$ be an m-vector equal to the first derivative of the penalty function, and let $\mathbf{J}_{pos}$ contain the positive elements of $\mathbf{J}$, and $\mathbf{J}_{neg}$ contain the negative elements of $\mathbf{J}$, so that $\mathbf{J} = \mathbf{J}_{pos} - \mathbf{J}_{neg}$. We can compute two m-vectors $\mathbf{s}_{pos}$ and $\mathbf{s}_{neg}$ so that $\mathbf{s}_{pos} = \mathbf{p}^\top \mathbf{f} \boldsymbol{\delta} + \mathbf{p}^{*\top} \boldsymbol{\delta}_{pos}^* \mathbf{e} - \mathbf{J}_{neg}$ and $\mathbf{s}_{neg} = \mathbf{p}^{*\top} \boldsymbol{\delta}_{neg}^* \mathbf{e} + \mathbf{p}_I^{*\top} \boldsymbol{\delta}_I^* \mathbf{e} + \mathbf{J}_{pos}$, where the ith element of $\boldsymbol{\delta}_{pos}^*$ is $\delta_i^L \frac{S(y_i)}{1 - S(y_i)} + \delta_i^I \frac{S(t_i^R)}{S(t_i^L) - S(t_i^R)}$ and the ith element of $\boldsymbol{\delta}_{neg}$ is $\delta_i + \delta_i^R$. We can then compute $\mathbf{s} = \mathbf{s}_{pos} - \mathbf{s}_{neg}$. Finally, we can compute $\mathbf{g}_\theta$, an $m \times m$ diagonal matrix with $(u, u)^{th}$ element $\theta_u/(s_{neg,u} + \xi)$, where ξ is a small constant included to avoid a zero denominator in the computation of $\mathbf{g}_\theta$.

2 Asymptotic consistency and normality proofs

In this section we state asymptotic consistency and normality results that allow for large-sample inferences to be made on regression parameters and survival quantities from the proposed model. Importantly, these results mean that our method does not require the use of computationally intensive methods such as bootstrapping for inference.

2.1 Asymptotic consistency

For the asymptotic consistency result, we denote the true values of the parameters $\boldsymbol{\beta}$, $\boldsymbol{\gamma}$ and $h_0(t)$ as $\boldsymbol{\beta}_0$, $\boldsymbol{\gamma}_0$ and $h_{00}(t)$. Let a and b be the minimum and maximum of all the observed survival times respectively, including interval censoring but excluding 0 and ∞. Then, let $C^r[a, b]$ be the set of functions that have r continuous derivatives over $[a, b]$. The parameter space for $\boldsymbol{\beta}$ can be given by $B = \{\boldsymbol{\beta} : |\beta_k| \leq C_1 < \infty, \forall k\}$. The parameter space for $\boldsymbol{\gamma}$ can

be given by $G = \{\boldsymbol{\gamma} : |\gamma_j| \leq C_2 < \infty, \forall j\}$. The parameter space for $h_0(t)$ can be given by $A = \{h_0(t) : h_0 \in C^r[a,b], 0 \leq h_0(t)] \leq C_3 < \infty, \forall t \in [a,b]\}$. Therefore, the parameter space for $\boldsymbol{\tau} = (\boldsymbol{\beta}, \boldsymbol{\gamma}, h_0(t))$ is $\boldsymbol{\Gamma} = \{\boldsymbol{\tau} : \boldsymbol{\beta} \in B, \boldsymbol{\gamma} \in G, h_0 \in A\}$. Before defining the MPL estimator of $\boldsymbol{\tau}$, it is necessary to account for the fact that this method estimates an approximation of $h_0(t)$. For convenience, the approximation can be denoted as $\tilde{h}_0(t) = \sum_{u=1}^{m} \theta_u \psi_u(t)$. The parameter space for $\tilde{h}_0(t)$ can be given by $A_n = \{\tilde{h}_0(t) : 0 \leq \tilde{h}_0(t) \leq C_4 < \infty, \forall t \in [a,b]\}$. Then the parameter space for $\boldsymbol{\tau}_n$ is $\boldsymbol{\Gamma}_n = \{\boldsymbol{\tau}_n : \boldsymbol{\beta} \in B, \boldsymbol{\gamma} \in G, \tilde{h}_0 \in A_n\}$. The MPL estimator of $\boldsymbol{\tau}_n$ is then $\hat{\boldsymbol{\tau}}_n = (\hat{\boldsymbol{\beta}}, \hat{\boldsymbol{\gamma}}, \hat{h}_0(t))$. We require the following conditions:

A1. The matrices $\mathbf{X}$ and $\mathbf{Z}$ are bounded, and both $E(\mathbf{X}\mathbf{X}^T)$ and $E(\mathbf{Z}\mathbf{Z}^T)$ are non-singular.

A2. The penalty function $J(\boldsymbol{\eta})$ is bounded over $\boldsymbol{\Gamma}$ and $\boldsymbol{\Gamma}_n$.

A3. For function $\tilde{h}_0(t)$, there is a constant C_5 independent of n that is the upper bound of all $\theta_u \geq 0$. Additionally, the basis functions $\psi_u(t)$, where $u = 1, ..., m$, are bounded for $t \in [a,b]$.

A4. The knots and basis functions are selected such that for any $h_0(t) \in A$ there is a $\tilde{h}_0(t) \in A_n$ which satisfies $\max_t |\tilde{h}_0(t) - h_0(t)| \to 0$ when $n \to \infty$.

Theorem 1 demonstrates asymptotic consistency for $\hat{\boldsymbol{\tau}}_n$ when the number of basis functions $m \to \infty$ but $m/n \to 0$ when $n \to \infty$, and the scaled smoothing value $\mu_n = \lambda/n \to 0$ when $n \to \infty$. For proofs we refer the reader to **?**.

Theorem 1. *Assume that conditions A1-A4 hold. Assume that $h_0(t)$ is bounded and has some number $r \geq 1$ derivatives over the interval $[a,b]$. Assume that $m = n^v$, where $0 < v < 1$. Then, when $n \to \infty$,*

1. $||\hat{\boldsymbol{\beta}} - \boldsymbol{\beta}_0|| \to 0$ *almost surely, and*
2. $||\hat{\boldsymbol{\gamma}} - \boldsymbol{\gamma}_0|| \to 0$ *almost surely, and*
3. $\sup_{t \in [a,b]} |\hat{h}_0(t) - h_{00}(t)| \to 0$ *almost surely.*

2.2 Asymptotic normality

For the asymptotic normality results, we comment that it is necessary to now restrict m to be a finite number, as in **?** and **?**. We note however that the fixed m is not pre-determined as it depends on the sample size n; we use $m = n_0^{1/3}$ as a rough guide, where n_0 is the non-right censored sample size. In addition, for the asymptotic normality results we require a method to take into account potentially active constraints in the estimation of $\boldsymbol{\theta} \geq 0$. These active constraints are particularly likely to occur where there is a larger number of knots specified than strictly necessary, as the smoothing parameter will push these unnecessary parameters to 0. Ignoring active constraints may lead to negative variance in the estimation of $\boldsymbol{\theta}$.

To address the possible presence of active constraints, we adopt the following method. Recall that we can denote $\boldsymbol{\eta} = [\boldsymbol{\beta}, \boldsymbol{\gamma}, \boldsymbol{\theta}]^\top$, the parameter vector with length $p + q + m$. Let the

MPL estimate of $\boldsymbol{\eta}$ be $\hat{\boldsymbol{\eta}}$, and let $\boldsymbol{\eta}_0$ be the true value of the parameter vector. Without loss of generality, we assume that the first r values of $\boldsymbol{\theta}$ are 0, and as such are actively constrained. Define

$$\mathbf{U} = [\mathbf{0}_{(m-r+p+q)\times r}, \mathbf{I}_{(m-r+p+q)\times(m-r+p+q)}] \tag{1}$$

where $\mathbf{0}$ is a matrix of zeroes and $\mathbf{I}$ is an identity matrix. We have the condition $\mathbf{U}^\top\mathbf{U} = \mathbf{I}_{(m-r+p+q)\times(m-r+p+q)}$ satisfied. We have the conditions:

B1. The distributions of $\mathbf{x}_i$ and $\mathbf{z}_i$ are independent of $\boldsymbol{\eta}$.

B2. The limit $\lim_{n\to\infty}[n^{-1}l(\boldsymbol{\eta})]$ exists and has a unique maximum at $\boldsymbol{\eta}_0 \in \Omega$, where Ω is the parameter space for $\boldsymbol{\eta}$ and is a compact subspace of $\mathbf{R}^{p+q+m}$. That is to say, if the sample size is infinity, the true parameters can be obtained exactly from maximising the likelihood.

B3. $l(\boldsymbol{\eta})$ has a finite upper bound and is twice continuously differentiable in a neighbourhood of $\boldsymbol{\eta}_0$, and the matrices

$$\lim_{n\to\infty} n^{-1}\sum_{i=1}^{n}\frac{\partial l_i(\boldsymbol{\eta})}{\partial\boldsymbol{\eta}}\frac{\partial l_i(\boldsymbol{\eta})}{\partial\boldsymbol{\eta}^T}$$

and

$$\lim_{n\to\infty}\left[-n^{-1}\frac{\partial^2(\boldsymbol{\eta})}{\partial\boldsymbol{\eta}\partial\boldsymbol{\eta}^T}\right]$$

exist.

B4. The penalty function $J(\boldsymbol{\eta})$ is twice continuously differentiable on Ω, and these derivatives are bounded.

B5. The matrix $\mathbf{U}^T\mathbf{F}(\boldsymbol{\eta})\mathbf{U}$ is invertible in a neighbourhood of $\boldsymbol{\eta}_0$.

Theorem 2 states the asymptotic normality results under these conditions; we again refer to **?** for detailed proofs.

Theorem 2. *Let $\mu_n = \lambda/n$. Assume that $\mu_n = o(n^{1/2})$ and that we have the first r active constraints in the MPL estimate of $\boldsymbol{\theta}$. Define matrix $\mathbf{U}$ as above. Assume B1-B5 hold. Let*

$$\mathbf{F}(\boldsymbol{\eta}) = -E_{\boldsymbol{\eta}_0}\left[\lim_{n\to\infty} n^{-1}\frac{\partial^2 l(\boldsymbol{\eta})}{\partial\boldsymbol{\eta}\partial\boldsymbol{\eta}^T}\right].$$

Under these conditions, when $n \to \infty$, $\sqrt{n}(\hat{\boldsymbol{\eta}} - \boldsymbol{\eta}_0)$ converges in distribution to $\mathcal{N}(\mathbf{0}, \tilde{\mathbf{F}}(\boldsymbol{\eta}_0)^{-1})$, where $\tilde{\mathbf{F}}(\boldsymbol{\eta}_0)^{-1} = \mathbf{U}(\mathbf{U}^T\mathbf{F}(\boldsymbol{\eta})\mathbf{U})^{-1}\mathbf{U}^T$.

To implement the results of Theorem 2, we require a method for identifying the presence of active constraints in practice. The method used here closely follows that proposed by **?**. Active constraints can be identified by inspecting both the value of $\hat{\theta}_u$ and the corresponding gradient for each u. After the Newton-MI algorithm has reached convergence, some $\hat{\theta}_u$ may be exactly zero with negative gradients, and thus are clearly subject to an active constraint. Furthermore, there may be some $\hat{\theta}_u$ that are very close to, but not exactly, zero. For these $\hat{\theta}_u$,

a corresponding negative gradient value is indicative that they are also subject to an active constraint. In practice, active constraints are defined where, for a given u, $\hat{\theta}_u < 10^{-3}$ and the corresponding gradient is less than $-\varepsilon$ where ε is a positive threshold value such as 10^{-2}. After the indices associated with active constraints are identified, obtaining the matrix $\tilde{\mathbf{F}}(\boldsymbol{\eta}_0)^{-1}$ is a very straightforward computation. The matrix $\mathbf{U}^T\mathbf{F}(\boldsymbol{\eta})\mathbf{U}$ is obtained by removing the rows and columns of $\mathbf{F}(\boldsymbol{\eta})$ associated with the active constraints. The result is then inverted, and then padded with zeros in the deleted rows and columns to obtain $\tilde{\mathbf{F}}(\boldsymbol{\eta}_0)^{-1}$. To make use of these asymptotic results for inference on finite samples, it is necessary to approximate the distribution for $\hat{\boldsymbol{\eta}}$ when n is large. Doing so also incorporates non-zero values for the smoothing parameter λ into the inference on the parameter estimates. The necessary results are presented below in Corollary 1.

Corollary 1. *Assume that the smoothing parameter $\lambda \ll n$. Define*

$$\mathbf{A}(\hat{\boldsymbol{\eta}})^{-1} = \mathbf{U}\left(\mathbf{U}^T\left(\frac{\partial^2 l(\hat{\boldsymbol{\eta}})}{\partial\boldsymbol{\eta}\partial\boldsymbol{\eta}^T} + \lambda\frac{\partial^2 J(\hat{\boldsymbol{\eta}})}{\partial\boldsymbol{\eta}\partial\boldsymbol{\eta}^T}\right)\mathbf{U}\right)^{-1}\mathbf{U}^T$$

Then, when n is large, the distribution for the MPL estimate $\hat{\boldsymbol{\eta}} - \boldsymbol{\eta}_0$ can be approximated by a multivariate normal distribution having mean zero and covariance matrix

$$\widehat{var}(\hat{\boldsymbol{\eta}}) = \mathbf{A}(\hat{\boldsymbol{\eta}})^{-1}\frac{\partial^2 l(\hat{\boldsymbol{\eta}})}{\partial\boldsymbol{\eta}\partial\boldsymbol{\eta}^T}\mathbf{A}(\hat{\boldsymbol{\eta}})^{-1}$$

These results allow for inferences to be made not only on both sets of regression parameters but also on quantities associated with the baseline hazard function.

3 Additional simulation results

Table 1: Study 1 (right censoring): Regression parameters of MPL and PL methods with exponential baseline for $n = 200$ and $n = 1000$.

		$n = 200$						$n = 1000$					
		$\pi^E = 0.7$			$\pi^E = 0.3$			$\pi^E = 0.7$			$\pi^E = 0.3$		
		Bias	SE	CP	Bias	SE	CP	Bias	SE	CP	Bias	SE	CP
β_1	MPL	0.0221	0.1648	0.95	0.0599	0.2322	0.89	0.0209	0.0732	0.93	-0.0105	0.1049	0.88
			(0.1637)			(0.2870)			(0.0706)			(0.1662)	
	PL	-0.1429	0.1523	0.80	-0.6798	0.1453	0.01	-0.1472	0.0667	0.42	-0.7065	0.0601	0.00
			(0.1700)			(0.1482)			(0.0743)			(0.0714)	
β_2	MPL	0.0212	0.2610	0.89	0.0700	0.3567	0.87	0.0236	0.1162	0.95	0.0164	0.1608	0.92
			(0.3230)			(0.4684)			(0.1169)			(0.1754)	
	PL	0.0963	0.2519	0.91	0.3506	0.2511	0.70	0.1043	0.1110	0.89	0.3328	0.1094	0.13
			(0.2817)			(0.2607)			(0.1120)			(0.1011)	
γ_1	MPL	0.0272	0.1839	0.95	0.0244	0.3255	0.95	0.0204	0.0839	0.97	0.0112	0.1536	0.92
			(0.3019)			(0.4199)			(0.1331)			(0.1844)	
	PL	-0.0123	0.2782	0.94	-0.3792	0.2088	0.60	0.0119	0.1228	0.97	-0.4042	0.0806	0.00
			(0.2977)			(0.2076)			(0.1400)			(0.0821)	

Table 2: Study 1: Baseline survival function results with exponential baseline for $n = 200$ and $n = 1000$.

		$n = 200$						$n = 1000$					
		$\pi^E = 0.7$			$\pi^E = 0.3$			$\pi^E = 0.7$			$\pi^E = 0.3$		
		Bias	SE	CP	Bias	SE	CP	Bias	SE	CP	Bias	SE	CP
t_1	MPL	-0.0005	0.0172	0.94	0.0007	0.0118	0.95	0.0015	0.0073	0.94	0.0003	0.0047	0.95
	PL	0.0075	0.0134	0.92	-0.0062	0.0119	0.92	0.0085	0.0069	0.73	-0.0053	0.0054	0.86
t_2	MPL	0.0009	0.0490	0.95	0.0040	0.0382	0.97	0.0068	0.0186	0.91	0.0008	0.0142	0.94
	PL	0.0268	0.0325	0.88	-0.1495	0.0344	0.03	0.0305	0.0144	0.42	-0.0196	0.0105	0.51
t_3	MPL	0.0088	0.0870	0.96	0.0189	0.0710	0.97	0.0127	0.0369	0.93	0.0001	0.0257	0.95
	PL	0.0489	0.0664	0.87	-0.4127	0.0464	0.00	0.0551	0.0320	0.60	-0.3379	0.0211	0.00

Table 3: Study 2, Scenario 1 (partly-interval censoring): Regression parameters of MPL method with log-logistic baseline hazard function for $n = 200$ and $n = 1000$.

	$n = 200$						$n = 1000$					
	$\pi^E = 0.7$			$\pi^E = 0.3$			$\pi^E = 0.7$			$\pi^E = 0.3$		
	Bias	SE	CP	Bias	SE	CP	Bias	SE	CP	Bias	SE	CP
β_1	-0.0042	0.1842	0.94	0.0041	0.2086	0.99	0.0126	0.0743	0.96	-0.0025	0.0894	0.96
β_2	0.0974	0.2900	0.93	0.0923	0.3271	0.98	0.0152	0.1383	0.96	0.0438	0.1383	0.92
γ_1	0.0931	0.3143	0.94	0.1820	0.3708	0.98	0.0935	0.1314	0.89	0.0866	0.1672	0.92

Table 4: Study 2, Scenario 1 (partly-interval censoring): Baseline survival function results from MPL method with log-logistic baseline hazard function for $n = 200$ and $n = 1000$.

	$n = 200$						$n = 1000$					
	$\pi^E = 0.7$			$\pi^E = 0.3$			$\pi^E = 0.7$			$\pi^E = 0.3$		
	Bias	SE	CP	Bias	SE	CP	Bias	SE	CP	Bias	SE	CP
t_1	0.0401	0.1602	1.00	0.0725	0.1978	1.00	0.0210	0.0795	1.00	0.0220	0.1078	1.00
t_2	0.0169	0.0608	0.96	0.0589	0.1281	0.96	0.0048	0.0140	0.97	0.0045	0.0221	0.97
t_3	0.0096	0.0291	0.94	0.0405	0.0798	0.94	0.0013	0.0034	0.94	0.0013	0.0058	0.97

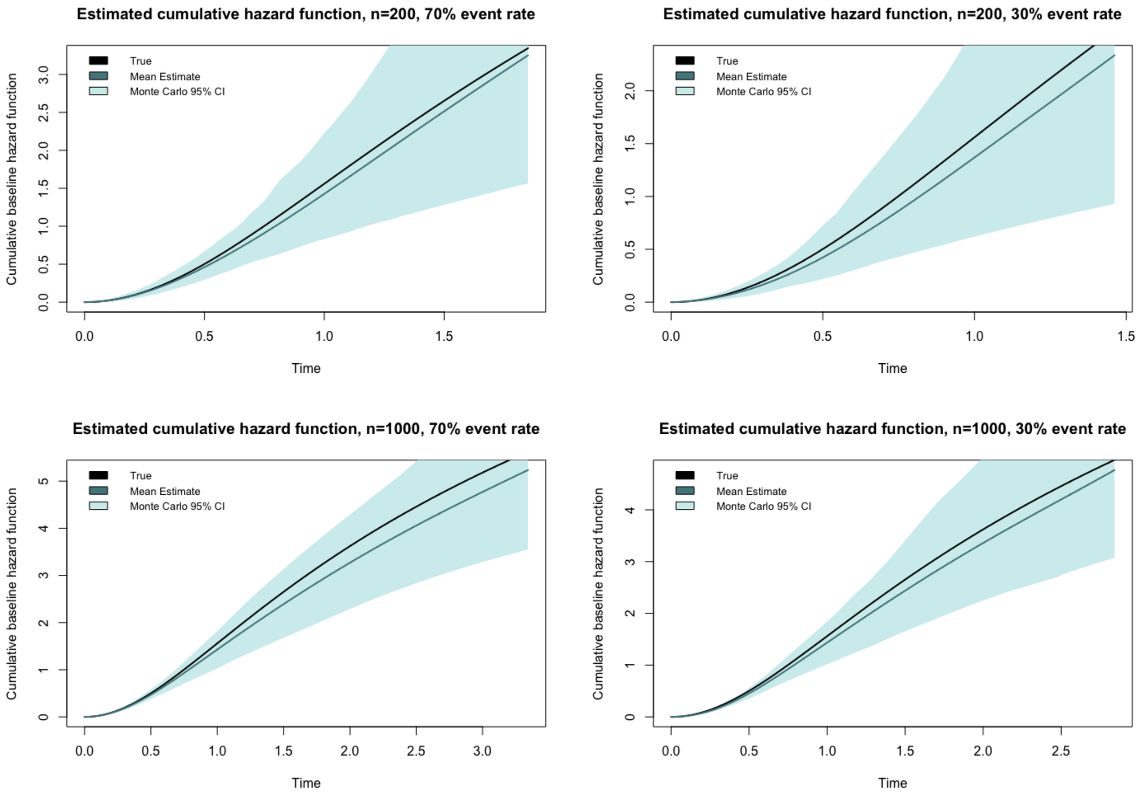

Figure 1: Study 2, Scenario 1: True vs. estimated baseline cumulative hazard function for log-logistic baseline hazard function.

Table 5: Study 2, Scenario 2 (partly-interval censoring): Regression parameters of MPL and Transformation Model (TM) method for two discrete time-varying covariates, for $n = 200$ and $n = 1000$.

		$n = 200$			$n = 1000$		
		Bias	SE	CP	Bias	SE	CP
β_1	MPL	0.1034	0.1160	0.80	0.088	0.099	0.71
			(0.1371)			(0.125)	
	TM	0.1317	0.1274	0.76	0.141	0.056	0.23
			(0.1431)			(0.049)	
β_2	MPL	-0.1139	0.1584	0.88	-0.106	0.152	0.89
			(0.1415)			(0.127)	
	TM	-0.1165	0.1243	0.79	-0.139	0.052	0.30
			(0.1570)			(0.056)	
γ_1	MPL	0.0894	0.3357	0.99	0.136	0.324	0.99
			(0.2016)			(0.174)	
	TM	0.2022	0.2491	0.88	0.194	0.115	0.68
			(0.3062)			(0.108)	
γ_2	MPL	-0.0395	0.3637	0.95	-0.045	0.138	0.63
			(0.3891)			(0.243)	
	TM	-0.1137	0.2535	0.93	-0.309	0.111	0.21
			(0.2541)			(0.108)	

Table 6: Study 2, Scenario 2 (partly-interval censoring): Cumulative hazard function results from MPL and Transformation Model (TM) method for two discrete time-varying covariates, for $n = 200$ and $n = 1000$.

		$n = 200$			$n = 1000$		
		Bias	SE	CP	Bias	SE	CP
t_1	MPL	0.005	0.010	0.97	0.006	0.008	0.94
	TM	0.001	0.022	0.99	-0.255	0.011	0.00
t_2	MPL	0.026	0.043	0.94	0.109	0.028	0.05
	TM	0.012	0.064	0.99	-0.399	0.036	0.00
t_3	MPL	0.066	0.111	0.93	0.206	0.062	0.09
	TM	0.025	0.133	0.99	-0.588	0.059	0.00

Table 7: Study 2, Scenario 1 sub-study (partly-interval censoring, unbalanced values in Z_1): Regression parameters of MPL method with Weibull baseline hazard function for $n = 200$ and $n = 1000$.

	$n = 200$						$n = 1000$					
	$\pi^E = 0.7$			$\pi^E = 0.3$			$\pi^E = 0.7$			$\pi^E = 0.3$		
	Bias	SE	CP	Bias	SE	CP	Bias	SE	CP	Bias	SE	CP
β_1	0.0139	0.1500	0.92	-0.0060	0.1787	0.93	-0.0078	0.0665	0.93	-0.0050	0.0783	0.92
		(0.1706)			(0.1966)			(0.0719)			(0.0850)	
β_2	0.0408	0.2166	0.85	0.0031	0.2371	0.85	-0.0169	0.0970	0.92	-0.0474	0.1044	0.85
		(0.2728)			(0.3327)			(0.1125)			(0.1708)	
γ_1	-0.0188	0.3488	0.80	-0.0437	0.6319	0.90	0.0114	0.2373	0.94	0.0151	0.2708	0.91
		(0.5426)			(1.4136)			(0.2498)			(0.3342)	

Table 8: Study 2, Scenario 1 sub-study (partly-interval censoring, unbalanced values in Z_1): Baseline survival function results from MPL method with Weibull baseline hazard function for $n = 200$ and $n = 1000$.

	$n = 200$						$n = 1000$					
	$\pi^E = 0.7$			$\pi^E = 0.3$			$\pi^E = 0.7$			$\pi^E = 0.3$		
	Bias	SE	CP	Bias	SE	CP	Bias	SE	CP	Bias	SE	CP
t_1	0.0006	0.0268	1.00	0.0005	0.0276	1.00	-0.0031	0.0256	1.00	-0.0001	0.0275	1.00
t_2	0.0136	0.1130	1.00	-0.0028	0.1291	1.00	-0.0046	0.0861	1.00	-0.0127	0.1040	1.00
t_3	0.0041	0.0931	1.00	-0.0042	0.1366	1.00	-0.0015	0.0377	1.00	-0.0033	0.0636	1.00
